# Evolutionary trends in the elasmobranch neurocranium

**DOI:** 10.1038/s41598-024-62004-3

**Published:** 2024-05-20

**Authors:** Joel H. Gayford, Martin D. Brazeau, Gavin J. P. Naylor

**Affiliations:** 1 Department of Life Sciences, Silwood Park Campus, Imperial College London, London, UK; 2 Shark Measurements, London, UK; 3 The Natural History Museum, Cromwell Road, London, SW7 5BD UK; 4 Florida Museum of Natural History, University of Florida, Gainesville, FL USA

**Keywords:** Ecology, Evolution

## Abstract

The neurocranium (braincase) is one of the defining vertebrate characters. Housing the brain and other key sensory organs, articulating with the jaws and contributing to the shape of the anteriormost portion of the body, the braincase is undoubtedly of great functional importance. Through studying relationships between braincase shape and ecology we can gain an improved understanding of form-function relationships in extant and fossil taxa. Elasmobranchii (sharks and rays) represent an important case study of vertebrate braincase diversity as their neurocranium is simplified and somewhat decoupled from other components of the cranium relative to other vertebrates. Little is known about the associations between ecology and braincase shape in this clade. In this study we report patterns of mosaic cranial evolution in Elasmobranchii that differ significantly from those present in other clades. The degree of evolutionary modularity also differs between Selachii and Batoidea. In both cases innovation in the jaw suspension appears to have driven shifts in patterns of integration and modularity, subsequently facilitating ecological diversification. Our results confirm the importance of water depth and biogeography as drivers of elasmobranch cranial diversity and indicate that skeletal articulation between the neurocranium and jaws represents a major constraint upon the evolution of braincase shape in vertebrates.

## Introduction

The analysis of morphological shape can be used to investigate key aspects of animal life history relevant to their ecology and evolution. In recent years, authors have employed morphometrics of vertebrate neurocrania (braincases) to investigate implied patterns of morphological constraint and functional evolution^1–4^ in several large vertebrate clades. Knapp et al.^3^ recently conducted a pioneering study of the morphometrics of teleost fish braincases, focussed specifically on Pelagiaria. Noting that nearly all such studies to date had focussed on tetrapods (terrestrial vertebrates), they addressed a major gap in morphometric studies of vertebrate crania. The omission of fishes excluded approximately 50% of vertebrate diversity, limiting the kinds of generalities that could be drawn from such studies. In the present study, we add to this growing body of studies by exploring the patterns of morphological evolution in another key vertebrate group: the Elasmobranchii (sharks, skates, and rays).

The elasmobranch cranium is a potentially challenging but illustrative case study for morphological evolution for at least two reasons. Firstly, relative to other vertebrate crania it is comprised of few elements, largely consisting of a continuous cartilaginous unit. The lack of sutural boundaries between the major embryonic components renders potential module boundaries more ambiguous. Secondly, there is a high degree of anatomical decoupling between the neurocranium and the jaws^5^; the jaws have a highly kinetic connection to the neurocranium with mainly soft-tissue linkages. This looser biomechanical integration arguably predicts a lower degree of correlation between neurocranial shape and feeding mode. Moreover, there are numerous examples of cranial specialisation within Elasmobranchii^6,7^ that appear to have evolved to facilitate highly specialised ecologies^8,9^. Whilst the fossil preservation potential of the elasmobranch neurocranium is low, geometric information can often be recovered from some fossil chondrichthyans^10–12^. Thus, determining the ecomorphological relevance of neurocranial shape variation in extant forms can inform paleobiological interpretations, and aid in better understanding the paleoecology of extinct elasmobranch taxa. Unfortunately, existing ecomorphological studies of neurocranial evolution encompass less than 5% of extant elasmobranch diversity^5,13^. These studies are by no means representative of Elasmobranchii as a whole, and thus further studies incorporating additional data are necessary.

In this study we use a three-dimensional geometric morphometric approach to investigate the extent to which ecology and life history correlate with morphology in the elasmobranch braincase. To achieve this goal, we quantify shape variation in the elasmobranch neurocranium from a large, phylogenetically disparate computerised tomography (CT) scan data set. This study documents morphological variation within Elasmobranchii and potential form-function relationships which, if studied further, may facilitate the use of neurocranial shape information from fossils to infer the paleoecology of extinct taxa. As the first large-scale phylogenetic analysis of neurocranial shape variation in cartilaginous fishes this study provides the foundation for further comparative research of neurocranium ecomorphology across vertebrate diversity.

Such analyses will improve our understanding of vertebrate cranial evolution and elasmobranch morphological evolution, providing new insight into the factors underlying phenotypic disparity in this charismatic, ecologically important clade. This will also aid in determining how selection underpinning elasmobranch neurocranial morphology might compare to that acting upon other components of the cranium and in other clades.

## Methodology

### Data collection

**Figure 1:**
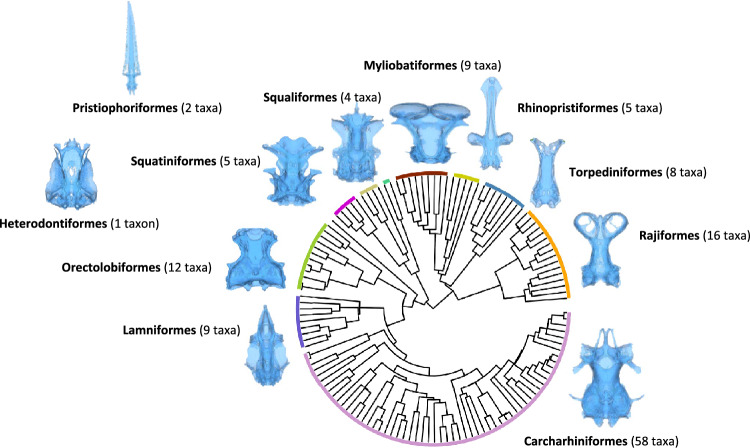
Phylogenetic composition of the dataset, using topology and branch lengths from Stein et al.^16^ based on data from Naylor et al.^17^ Blue outlines are neurocrania of representative taxa from each radiation in dorsal view.

The data set consisted of three-dimensional models of neurocrania of 130 species, representing most major elasmobranch radiations (Fig. 1). We obtained 112 segmented computerised tomography (CT) scans from the Chondrichthyan Tree of Life website^14^ and filled taxonomic gaps by incorporating a further 18 scans from Kamminga et al.^15^. We segmented these additional scans using Materialise Mimics [v25.0, https://www.materialise.com/en/healthcare/mimics-innovation-suite/mimics]. Specimen details and ecological data used can be found in the supplementary materials (Supplementary Table S1).

To investigate the relationship between ecology and neurocranial morphology, we gathered ecological data from FishBase^18^. We categorised biogeographic distribution qualitatively along a latitudinal gradient (boreal, temperate, subtropical, tropical) and quantitively (minimum, maximum, absolute median and absolute range values for latitude). We categorised water depth/habitat preference both qualitatively (demersal, bathydemersal, benthopelagic, bathypelagic, reef-associated, pelagic) and quantitatively (minimum, maximum, median and range values for water depth). We also included ‘water parameters’ (marine, freshwater, marine + brackish) as a qualitative descriptor of water chemistry preferences. We gathered qualitative data regarding reproductive mode (oviparous, ovoviviparous, viviparous) and conservation status (data deficient, least concern, near threatened, vulnerable, endangered, critically endangered, not evaluated), as well as a quantitative measure of body size (lm50, the length at which 50% of the population reaches sexual maturity). We categorised trophic ecology qualitatively as feeding mechanism (sensu Moss^18^; suction grasping, suction crushing, cutting, gouging, crushing, filter feeding), and quantitatively as trophic level (a value based on the fractional composition of diet sensu Cortés^19^). We also included dentition (sensu Cappetta^20^; clutching, clutching-grinding, crushing, cutting, cutting-clutching, tearing, filter feeding) as an additional qualitative measure relating to trophic ecology. Where certain ecological variables were absent for specific taxa, we excluded them from the relevant analyses. All of these ecological values for each taxon included in the study can be found in supplementary Table S1.

We extracted the phylogenetic tree topology and branch lengths (initially a set of 10,000 trees) used to correct subsequent phylogenetic analyses from Stein et al.^16^. During preliminary analyses a set of 100 trees (covering all major uncertainties in the Stein et al. phylogeny) was generated randomly from the full tree set, with each tree pruned to match the ecological dataset using the function match.phylo.data in package *picante*^21^ in the *R* statistical environment^22^. Preliminary analyses using these trees suggested that topology had negligible effect on results. Subsequently, a maximum clade credibility (MCC) tree was generated using in R for use in all analyses included in this study. This MCC tree was pruned using the approach described above and inspected prior to use to ensure that the pruning process did not substantially alter topology. See the data availability statement for code necessary to recreate this MCC topology.

**Figure 2:**
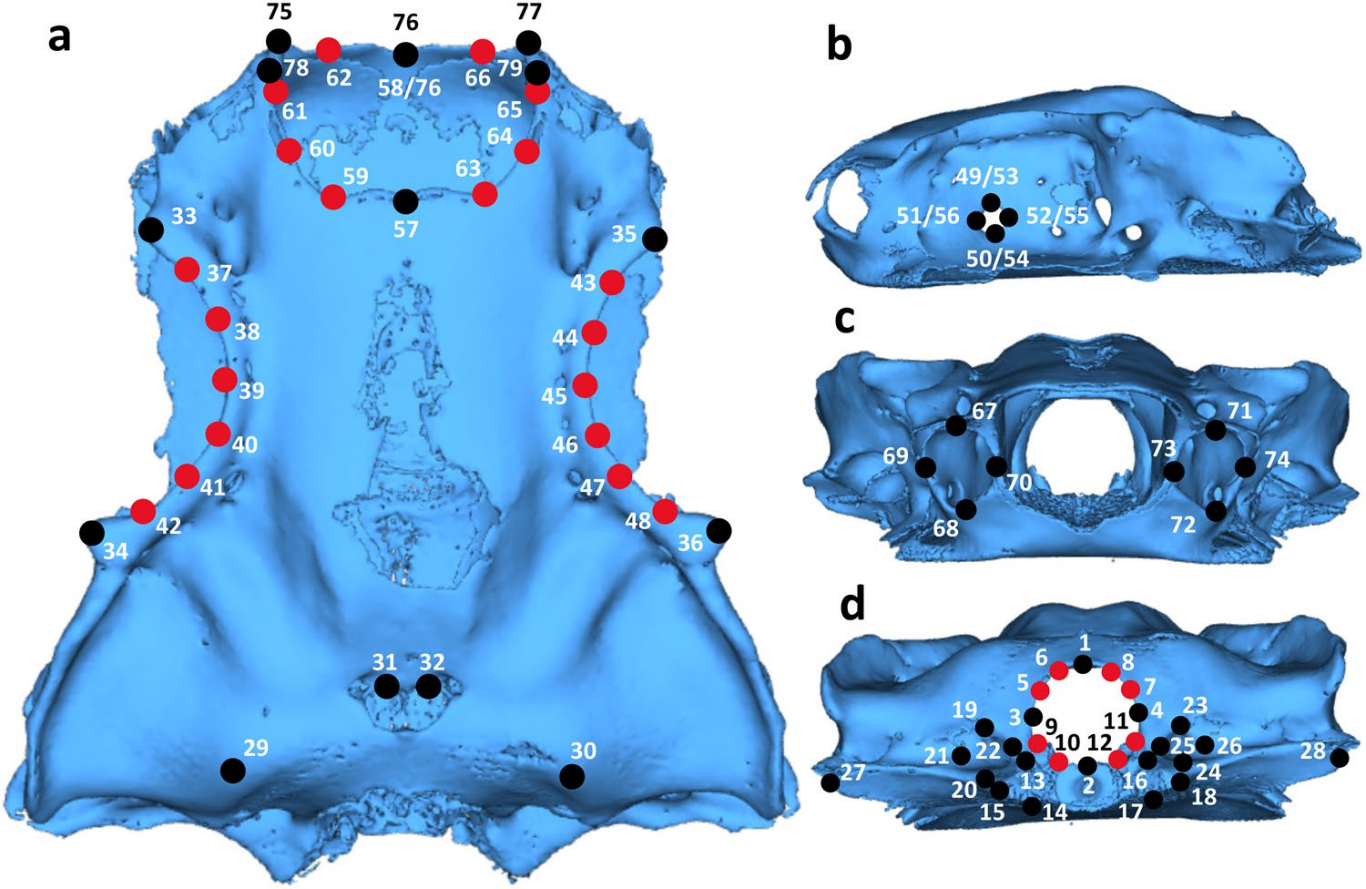
Landmarking scheme superimposed onto dorsal (**a**), left lateral (**b**), anterior (**c**), and posterior (**d**) views of *Sutorectus tentaculatus*. Numbers correspond to the order in which points were landmarked. Dual numbers 49–56 are those applied to left and right lateral views respectively. Dual numbers 58/76 result from the absence of a central rostral cartilage in this taxon, the anteriormost tip of which would otherwise represent number 76. Black points represent fixed landmarks whereas red points reflect sliding semilandmarks.

We defined a series of anatomical landmarks to optimise the trade-off between proportion of morphological variation captured and the number of taxa that could be incorporated into analyses. In selecting landmarks, we took a functionally informed approach, assigning landmarks to extremal points with likely functional significance. For instance, we sought to capture relative sizes and shapes of areas that house paired sensory capsules, as well as key attachment sites like the occiput, as well as summarizing the overall geometry in three dimensions. Thus, whilst some taxonomic groups included in this study lack structures present in others (e.g. the lack of preorbital processes in Torpediniformes) landmarks could still be assigned to the equivalent positions on the extremal surface of the neurocranium without being treated as missing data points as they delineate regional correspondence within the braincase. We identified anatomical points that could be reliably located in all specimens, excluding candidate landmarks that were not mineralised in a large number of specimens. Regions of the neurocranium that were not considered due to low variation and poor mineralisation include the majority of the nasal capsules and the ventral surface of the braincase. In total we selected 51 fixed landmarks (Bookstein type 1) and 28 sliding semilandmarks (Fig. 2). Landmark selection of this type is increasingly common, and the original requirement of strict biological homology of landmarks has been relaxed, instead favouring landmark selection that allows the testing of specific biological hypotheses^23,24^. Landmark definitions can be found in the supplementary materials (Supplementary Table S2). We collected landmark data using custom built landmark software associated with the Chondrichthyan Tree of Life Website^14^ and in the package *geomorph* in the R statistical environment^22,25^. Where a single landmark could not be located on a specimen due to poor specimen or scan quality (or poor mineralisation), we estimated its position using a ‘TPS’ method with the function estimate.missing in the package *geomorph* in the R statistical environment^22,25^. Details of missing landmarks can be found in Supplementary Table S1.

### Data analysis

We conducted all analyses in the R statistical environment^22^. To scale landmark data such that morphological differences are expressed in terms of shape alone, we performed generalised Procrustes analysis (GPA) using the package *geomorph*^25^. All subsequent analyses were performed upon the GPA-aligned landmark data.

To visualise the relationship between phylogeny and shape variation, we performed principal component analysis (PCA) and phylomorphospace reconstruction using the packages *phytools* and ggplot2^26,27^. We then tested for phylogenetic signal (measured as *K*_*mult*_, where values > 1 imply phylogenetic signal greater than expected under the assumption of Brownian motion) using the package *geomorph*^25^.

To search for relationships between neurocranium shape and ecology, we first performed a Procrustes ANOVA between Batoidea (skates and rays) and Selachii (sharks), between all extant elasmobranch orders (Rajiformes, Torpediniformes, Myliobatiformes, Rhinopristiformes, Orectolobiformes, Hexanchiformes, Heterodontiformes, Squaliformes, Squatiniformes, Pristiophoriformes, Lamniformes, Carcarhiniformes) and between bins for each of the qualitative ecological variables using the *geomorph* function procD.lm^25^. We repeated these analyses using phylogenetically informed Procrustes ANOVA to account for the influence of shared ancestry on morphology using the *geomorph* function procD.pgls^25^. Where we found significant (*p* < 0.05) results, we conducted pairwise comparisons using the *RRPP* function *pairwise*^28^. To test for differences in disparity (morphological variance), we input procD.pgls results into the *geomorph* function morphol.disparity^25^. Finally, we performed phylogenetic generalised least-squares (PGLS) regressions using the *geomorph* function procD.pgls^25^ to test for the effects of quantitative ecological variables upon neurocranium morphology.

To test for convergent evolution, we performed a k-means cluster analysis (dividing species into ‘morphotypes’) upon principal components summing to 95% of morphological variation using the *NbClust* function NbClust^29^, considering 2–15 possible clusters. We then calculated two different parameters describing the degree of convergence within these groups (C1 and Ct1) and compared their values to those which would be expected under 100 simulations of Brownian Motion evolution using the *convevol* functions convSig and convSigCt^30^.

We estimated the evolutionary history of the elasmobranch neurocranium by comparing Brownian Motion (BM), single-peak Ornstein Uhlenbeck (OU) and Early Burst (EB) models of trait evolution using the *mvMORPH* function mvgls^31^. We used generalised information criterion (GIC) values to compare between these models.

We tested for evolutionary integration/modularity using the *geomorph* functions phylo.modularity, phylo.integration and globalIntegration^25^. We tested six different hypotheses of modularity, ranging from 2 to 5 modules, adapting module partitions presented by López-Romero et al.^13^. Details of these partitions can be found in the supplementary materials (Supplementary Table S3). We repeated phylogenetically informed Procrustes ANOVA/PGLS analyses using the module partitions that received the most support in batoids and Selachii respectively to uncover differences in the ecological correlates of different portions of the braincase.

We estimated rates of morphological evolution (σ2, the multivariate rate of change for traits) using the geomorph function compare.multi.evol.rates^25^ between Batoidea and Selachii, between braincase modules, and between bins for each of the qualitative ecological variables collected. This approach determines the distance between taxa in morphospace following phylogenetic correction and uses this to calculate rates of shape variation and evolutionary rate ratios between traits. To determine whether rates between traits differ significantly, the observed rate is compared to a null value derived from 1000 simulations under Brownian Motion^32^.

All phylogenetically informed analyses assumed Brownian Motion evolution (traits evolve by accruing incremental changes drawn from a random distribution). Specimen size was not found to significantly influence neurocranium shape (*p* > 0.05) and was thus ignored as a potential covariate.

## Results

### Principal component analysis

**Figure 3:**
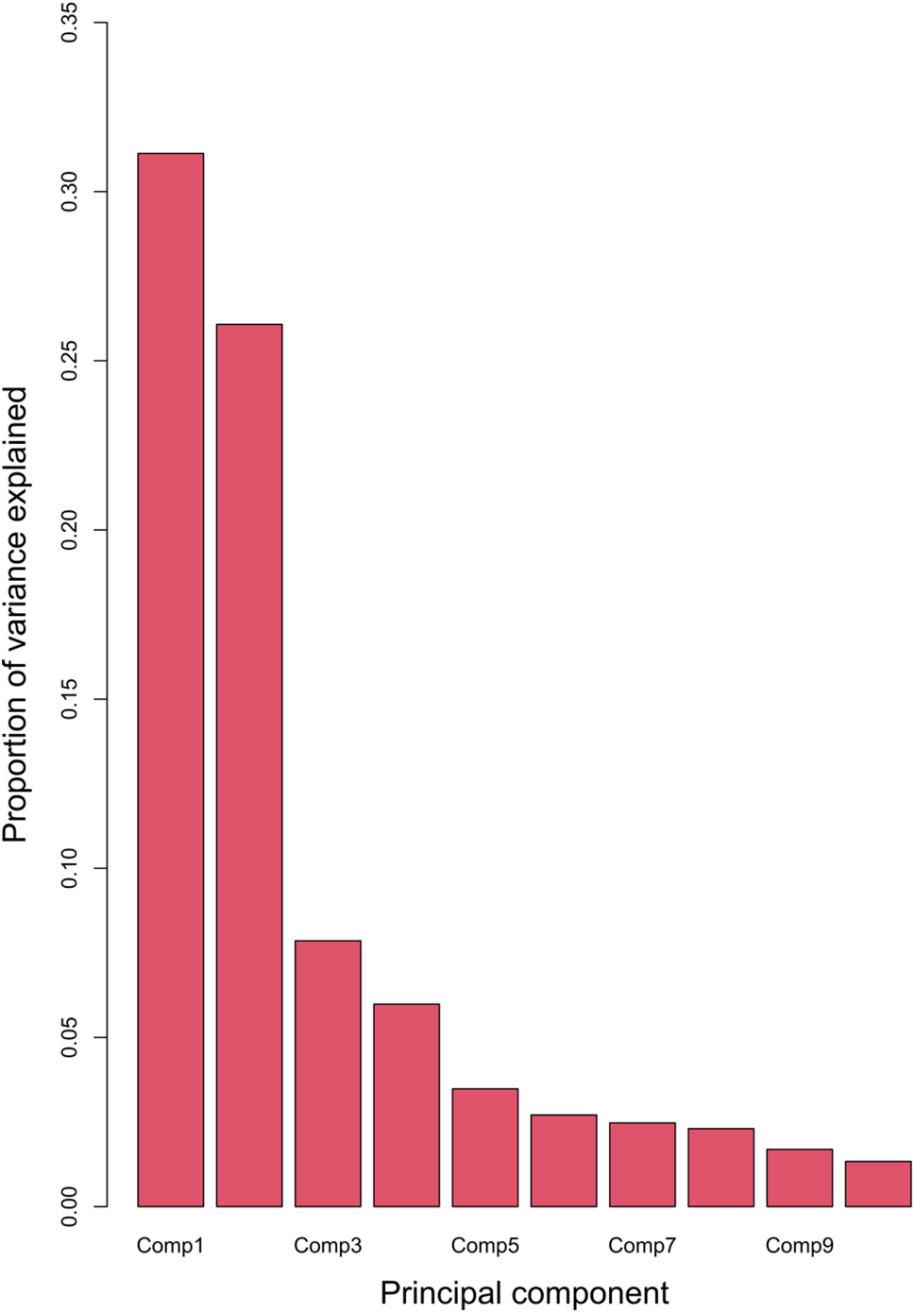
Screeplot showing that the first two principal components (Comp1-2) cumulatively explain 52.7% of shape variance, with no other principal component explaining over 10% of variance.

**Figure 4:**
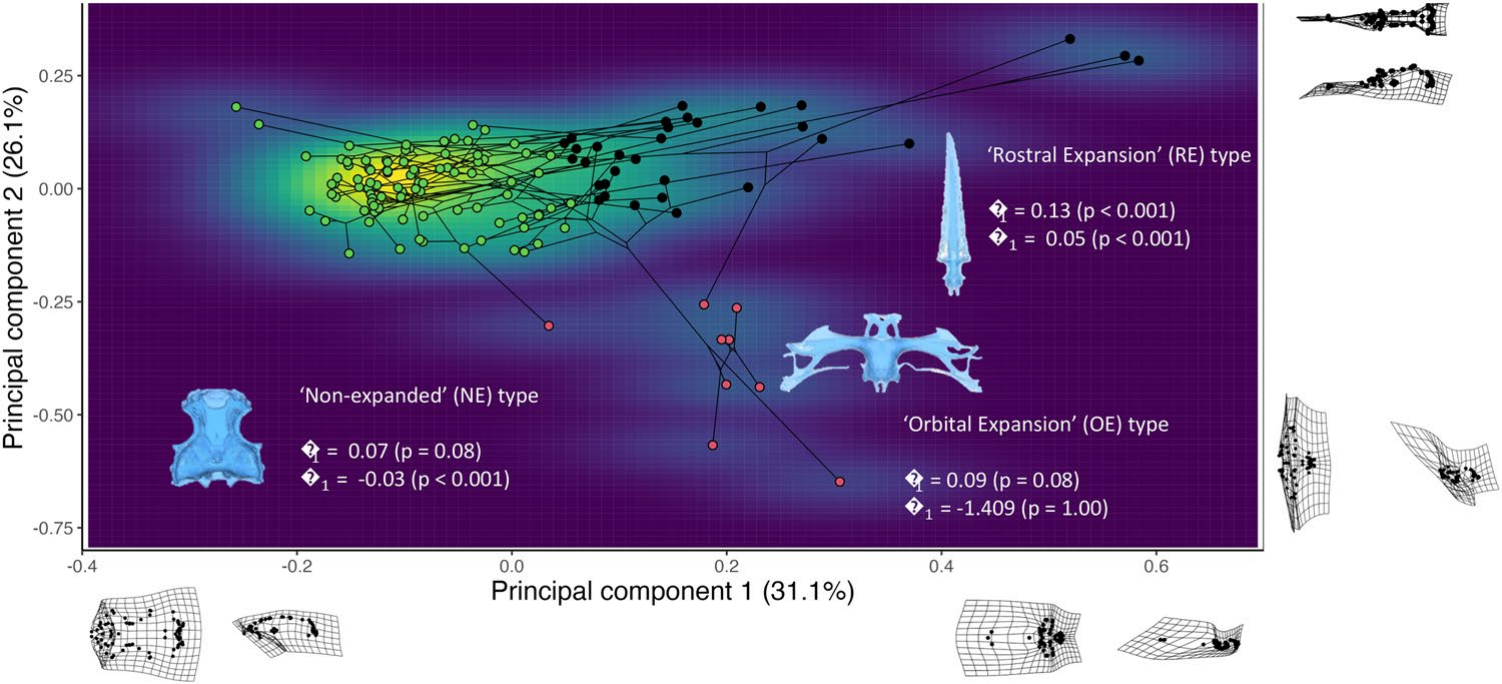
Phylomorphospace reconstruction showing morphospace occupancy density, overlain with the results of cluster analysis. Points are coloured by morphotype (as resolved through cluster analysis), alongside convergence values (C1 and Ct1) for each morphotype and a dorsal view of a representative taxon from said morphotype (blue neurocrania). Meshes reflect transformations of specimens with low and high values for principal components 1 and 2 respectively (*Sutorectus tentaculatus*, *Pliotrema warreni*, *Eusphyra blochii* and *Pristis clavata* respectively) upon the mean values of PC1 (dorsal meshes) and PC2 (lateral meshes). Note that meshes are not perfect representations of the morphological transitions represented by each PC, as each mesh has relatively extreme values for both PC1 and PC2 simultaneously. The distorted nature of some of these meshes results from high bending energy, associated with substantial movement of landmarks that are close together when comparing forms.

The first two principal components (PC) cumulatively explained 52.7% of shape variation, with no other PC explaining more than 10% of variation (Figs. 3,4). Examination of the relative warps allows qualitative assessment of the major shape differences along the PC axes. Principal component 1 (PC1) appears to relate to a morphocline ranging from broad, deep neurocrania with preorbital and postorbital regions roughly equivalent in size (negative values, e.g. *Hypnos monopterygius* and *Torpedo fuscomaculata*) to narrow and shallow neurocrania with a small preorbital region (positive values) such as *Pilotrema warreni* and *Pristiophorus nudipinnis* (Fig. 4). Principal component 2 (PC2) appears to relate to a morphocline ranging from neurocrania with a substantially laterally expanded orbital region and relatively small rostral cartilages (negative values, e.g. *Eusphyra blochii* and *Sphyrna lewini*) to narrow neurocrania with small/absent orbital processes and elongation of both the orbital and rostral regions (positive values) such as *Pristis clavata* or *Pristiophorus nudipinnis* (Fig. 4). Phylomorphospace reconstructions coloured by each qualitative ecological variable can be found in Supplementary Fig. S1-7.

### Phylogenetic signal

In each of Elasmobranchii $({K}_{mult} = 0.406)$, Batoidea $({K}_{mult} = 0.773)$, and Selachii $({K}_{mult} = 0.371)$, phylogenetic signal was weaker than expected under the expectations of Brownian motion (${K}_{mult}$<1), a result that was statistically significant in all cases $\left(p<0.001\right)$. Thus, phylogeny does not appear to be a major factor in explaining similarity in neurocranial shape. However, in light of the challenges associated with interpreting phylogenetic signal^33,34^, we undertook both phylogenetically corrected and un-corrected comparisons where appropriate.

### Non-phylogenetic ANOVA/GLS

MANOVA and GLS analysis recovered evidence of significant relationships $(p<0.05)$ between neurocranium shape and superorder, order, habitat, conservation status, reproductive mode, feeding type, dental type, body size, latitude (minimum, maximum, median and range) and water depth (maximum and range). Full output can be found in Supplementary Table S4.

### Phylogenetically informed ANOVA/PGLS

PMANOVA and PGLS analysis recovered evidence of significant association between neurocranium shape and water parameters (marine, brackish, freshwater), latitude (minimum, maximum, median and range) and water depth (maximum, median and range) (Table 1). Pairwise analysis of water parameter groups did not recover any significant differences between any two groups.

**Table 1:** Full PMANOVA and PGLS results for the overall neurocranium.

Covariate	Z	*p*
Superorder	− 3.26	0.99
Order	0.29	0.35
Habitat	0.73	0.22
Latitude (qualitative)	− 0.18	0.57
Conservation status	− 0.86	0.80
**Water parameters**	**2.91**	**0.01**
Reproductive mode	0.92	0.18
Feeding type	− 0.02	0.48
Dental type	0.29	0.35
Trophic level	1.14	0.13
Body size	1.50	0.07
**Latitude (minimum)**	**3.21**	** < 0.01**
**Latitude (maximum)**	**3.10**	**0.01**
**Latitude (median)**	**2.97**	** < 0.01**
**Latitude (range)**	**3.12**	**0.01**
Depth (minimum)	0.78	0.21
**Depth (maximum)**	**2.30**	**0.01**
**Depth (median)**	**1.91**	**0.02**
**Depth (range)**	**2.05**	**0.02**

Covariates found to have a significant correlation with neurocranium shape (*p* < 0.05) are highlighted in bold.

### Disparity

Pairwise analysis of morphological disparity (Procrustes variance) recovered evidence of significant differences in disparity between groups delineated by habitat, conservation status, water parameters, reproductive mode and dental type. Procrustes variance for each group can be found in Supplementary Table S5.

### Convergence and cluster analysis

Cluster analysis recovered three distinct morphotypes corresponding to areas of high morphospace occupancy (Fig. 4). Each of these morphotypes consists of both batoids and Selachii (Supplementary Table S1). The rostral expansion (RE) morphotype is characterised by relatively high values for both PC1 and PC2, with both the orbital and rostral regions exhibiting dorsoventral elongation relative to other taxa. The orbital processes are relatively small or absent. The orbital expansion (OE) morphotype is characterised by relatively high values for PC1 and moderate to low values for PC2. Rostral cartilages are present however the defining feature of this morphotype is an anteriorly broad neurocranium exhibiting substantial lateral elongation of the orbital region. The non-expanded (NE) morphotype is characterised by relatively low PC1 values and moderate to high PC2 values, with a broad neurocranium and small or absent rostral cartilages.

Convergence analysis comparing observed phenotypes to those generated by 100 simulations of BM evolution suggested significant convergence explaining between 5–13% of phenotypic variation in the RE morphotype (Fig. 4). There was no support for significance convergence underlying either the NE or OE morphotypes (Fig. 4).

### Models of trait evolution

Based on GIC values an OU model of trait evolution $(GIC= -11743)$ was favoured over both BM and EB models $(\Delta GIC\ge 257)$.

### Rates of morphological evolution

Across the neurocranium, superorder (rate ratio = 1.80, effect size = 3.96, *p* < 0.001), order (rate ratio = 11.72, effect size = 3.78, *p* < 0.001), habitat (rate ratio = 6.42, effect size = 3.22, *p* < 0.001), latitude (rate ratio = 3.90, effect size = 2.92, *p* < 0.001), conservation status (rate ratio = 10.00, effect size = 4.59, *p* < 0.001), water parameters (rate ratio = 9.30, effect size = 3.07, *p* < 0.001), reproductive mode (rate ratio = 2.34, effect size = 4.19, *p* < 0.001), feeding type (rate ratio = 9.85, effect size = 3.12, *p* < 0.001) and dental type (rate ratio = 4.84, effect size = 2.47, *p* < 0.001) were all found to be significantly associated with evolutionary rate. Specifically, the following results were obtained: Selachii exhibit higher evolutionary rate across the neurocranium than Batoidea and Pristiophoriformes, and Rhinopristiformes exhibited higher evolutionary rate than other orders. Reef-associated taxa exhibited higher evolutionary rate than those in other habitat categories and tropical taxa exhibited higher evolutionary rate than those in other latitude categories. Critically endangered taxa exhibited higher evolutionary rate than any other conservation status. Taxa capable of living in marine and brackish waters exhibited higher evolutionary rate than taxa restricted to marine or freshwater environments. Oviparous taxa exhibited higher evolutionary rate than viviparous or ovoviviparous taxa, and filter-feeding taxa exhibited higher evolutionary rate than all other groupings of dental and feeding type. Detailed results including the evolutionary rates of each group can be found in Supplementary Table S6.

### Modularity and integration

**Figure 5:**
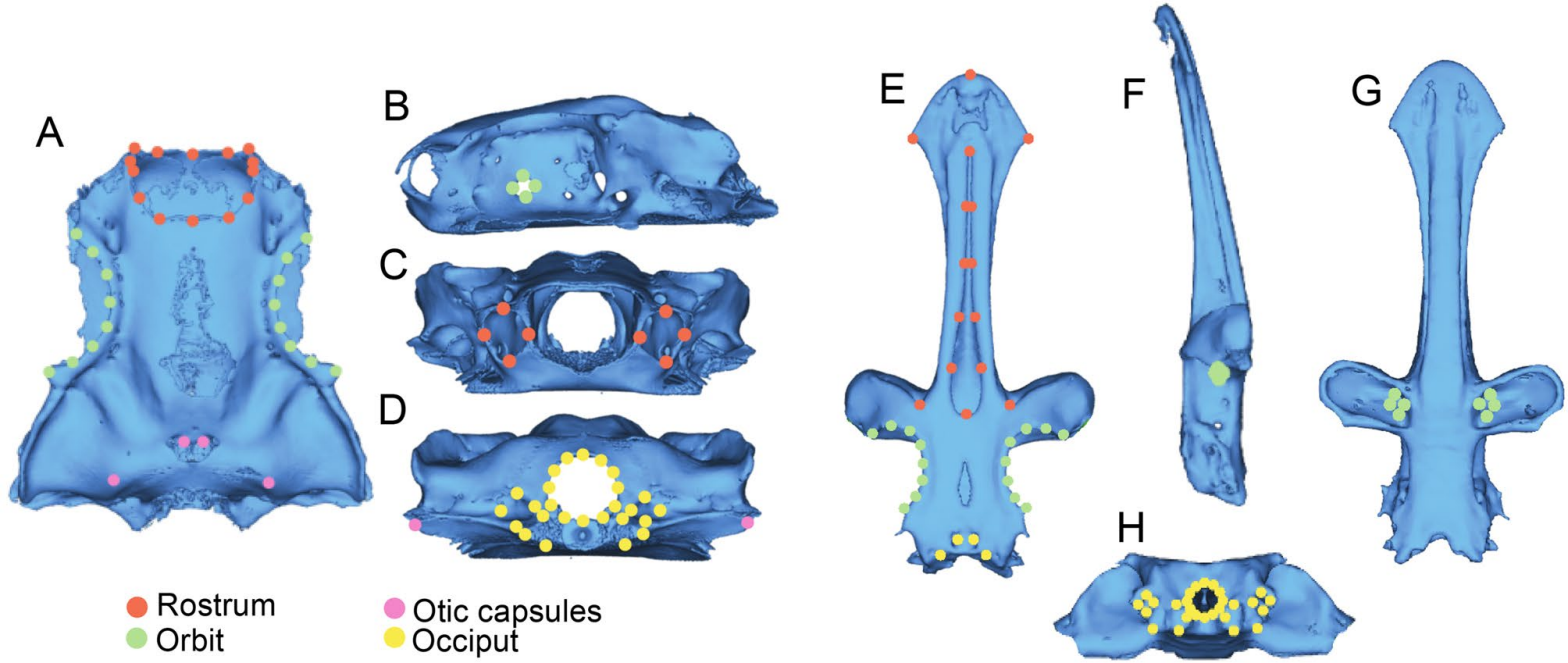
Dorsal, left lateral, anterior and posterior views of the neurocranium of *Sutorectus tentaculatus* (**a**–**d**) and *Pseudobatos lentiginosus* (**e**–**h**) displaying both landmarks and the modularity hypotheses best supported in Elasmobranchii and Selachii, and Batoidea, respectively. Coloration of a specific region of the neurocranium does not imply that this entire region falls under a given module, just that all landmarks located within this region do so.

Modularity analysis performed upon the full dataset provided greatest support for a three-module hypothesis (CR = 0.645, effect size = 3.97, *p* < 0.001; Fig. 5) defining the following modules: occipital (landmarks 1–32), orbit (landmarks 33–56 and 67–74), rostrum (landmarks 57–66 and 75–79). This analysis thus suggests the presence of a trait correlation between the occipital and otic regions of the neurocranium, and between the orbits and nasal capsules (Fig. 2; Supplementary Supplementary Table S2). Significant integration was identified between all modules $(rPLS: 0.70\le x\le 0.94, p<0.001)$ and across the entire neurocranium (global integration $= -1.43$). Whilst this finding may appear contradictory (as integration and modularity ostensibly represent alternative endpoints of a spectrum), this is not necessarily the case. Rather, these results suggest that there is some degree of integration between modules (as would be expected between anatomically connected subunits) but that integration within each module is stronger than the integration between modules. Full integration results can be found in Supplementary table S7. Significant differences in evolutionary rate between modules was found (rate ratio = 1.96, effect size = 1.91, *p* = 0.029) such that the rostral module (rate = 5.6e−6) has been evolving faster than either the orbital or occipital modules (rate = 3.90e−6).

Modularity analysis performed upon the batoid dataset provided greatest support for a four-module hypothesis (CR = 0.612, effect size = 8.03, *p* < 0.001; Fig. 5) defining the following modules: occipital (landmarks 1–26), otic (landmarks 27–32), orbit (landmarks 33–56), rostrum (landmarks 57–79). This analysis thus suggests the presence of a trait correlation between the rostrum and the nasal capsules (Fig. 2; Supplementary Table S2). Significant integration was identified between all modules $(rPLS: 0.57\le x\le 0.84, p<0.001)$ and across the entire neurocranium (global integration $= -1.43$). Significant differences in evolutionary rate between modules was found (rate ratio = 11.96, effect size = 4.08, $p<0.001$) such that the rostral module (rate = 4.82e−6) has been evolving faster than the orbital (rate = 2.29e−6) and otic (rate = 1.42e−6) modules, which have been evolving faster than the occipital module (rate = 4.03e−7).

Modularity analysis performed upon the Selachii dataset provided greatest support for the same hypothesis of modularity as supported in the full dataset (CR = 0.731, effect size = 4.88, *p* < 0.001; Fig. 5). Significant integration was identified between all modules $(rPLS: 0.78\le x\le 0.95, p<0.001)$ and across the entire neurocranium (global integration $= -1.40$). No significant difference in evolutionary rate between modules was recovered (rate ratio $= 1.43$, $p = 1.00$).

Module-based PMANOVA and PGLS analysis recovered evidence of significant correlations between several ecological covariates and subsets of neurocranial anatomy (Table 2). Full output can be found in Supplementary Table S8. Where relevant, pairwise analysis of groups delineated by water parameters, dental type, latitude and habitat failed to recover significant relationships (*p* $\ge$ 0.05). Relationships between quantitative variables and PC values can be found in the supplementary materials (Supplementary Figure S8–27).

**Table 2:** Statistically significant covariates arising from module-based PMANOVA and PGLS analyses.

Dataset	Module	Covariate	Z	*p*
Elasmobranchii	Occipital	Water parameters	2.87	0.01
		Latitude (minimum)	3.22	< 0.01
		Latitude (median)	2.52	< 0.01
		Latitude (maximum)	2.70	0.01
		Latitude (range)	3.16	0.01
		Depth (maximum)	1.92	0.02
		Depth (range)	1.78	0.03
	Orbit	Water parameters	2.52	0.01
		Body size	1.90	0.03
		Latitude (minimum)	2.66	< 0.01
		Latitude (median)	2.86	< 0.01
		Latitude (maximum)	2.83	< 0.01
		Latitude (range)	2.64	< 0.01
		Depth (median)	2.39	0.04
		Depth (maximum)	2.39	0.01
		Depth (range)	2.19	0.01
	Rostrum	Water parameters	2.86	0.01
		Latitude (minimum)	3.10	< 0.01
		Latitude (median)	2.74	< 0.01
		Latitude (maximum)	2.86	< 0.01
		Latitude (range)	3.03	< 0.01
		Depth (median)	2.03	0.02
		Depth (maximum)	2.10	0.01
		Depth (range)	1.76	0.04
Batoidea	Occipital	Latitude (qualitative)	1.98	0.02
		Latitude (minimum)	1.67	0.05
	Otic	Habitat	1.84	0.03
		Latitude (minimum)	2.07	0.02
		Latitude (median)	2.21	0.01
		Latitude (maximum)	2.06	0.02
	Orbit	Dental type	1.87	0.04
		Body size	2.37	< 0.01
	Rostrum	Depth (minimum)	1.66	0.05
Selachii	Occipital	Water parameters	3.20	< 0.01
		Trophic level	2.21	0.01
		Latitude (minimum)	3.05	0.01
		Latitude (median)	2.43	0.01
		Latitude (maximum)	2.72	0.01
		Latitude (range)	3.22	0.01
		Depth (maximum)	1.91	0.02
	Orbit	Water parameters	2.67	< 0.01
		Latitude (minimum)	2.54	0.01
		Latitude (median)	2.61	< 0.01
		Latitude (maximum)	2.64	0.01
		Latitude (range)	2.52	0.01
		Depth (maximum)	2.23	0.01
	Rostrum	Water parameters	3.19	0.01
		Latitude (minimum)	3.03	< 0.01
		Latitude (median)	2.73	< 0.01
		Latitude (maximum)	3.00	< 0.01
		Latitude (range)	3.02	< 0.01
		Depth (median)	1.82	0.03
		Depth (maximum)	2.29	0.01
		Depth (range)	2.31	0.01

Importantly only significant results (*p* < 0.05) are included, for full results including all tested covariates please refer to Supplementary Table S8.

## Discussion

The aim of this project was to explore morphological variation in the elasmobranch braincase using a diverse set of evolutionary analyses, and determine the extent to which braincase shape is a predictor of aspects of elasmobranch life-history and/or ecology in a phylogenetic context. Ecomorphological studies often reason that morphological shape variation under study has some functional significance. At the same time, functional and anatomical studies suggest that specific components of the elasmobranch braincase have different functions^35–37^. Comparative phylogenetic and morphometric analyses across the diversity of elasmobranchs can integrate these insights into a broader evolutionary picture.

We have uncovered several results that may be of evolutionary importance. Neurocranial shape variation is not evenly distributed across the braincase, but is concentrated in the orbits and rostrum, regions of the braincase which previous authors have suggested to be of high functional importance (Fig. 1)^35–37^. Rostrum geometry has obvious hydrodynamic consequences^35,37^, but also serves to support an anterior extension of the pectoral fin web and snout in batoids^36^. Orbit geometry can have profound variation in morphology with a range of functional correlates. These range from the extreme case of the cephalofoil in hammerheads^38^ to the role of the orbit wall as a major site of attachment for the upper jaw in squalomorphs^39,40^. Furthermore, orbit diameter and shape is a functional correlate of light conditions^41–43^ and therefore likely habitat occupation or life history, which has drawn interest in studies of fossil chondrichthyans^10^. We have found strong evidence for correlation between shape and ecology in the elasmobranch braincase—both at the level of the overall neurocranium, and that of individual ‘modules’ or subsets of neurocranial geometry (Tables 1,2). This, together with the results of modularity analyses, points towards the possible presence of independently evolving subunits within the neurocranium, that broadly correspond to the embryonic divisions of the skull—an observation that warrants further study (Fig. 5; Table 2).

In the absence of functional studies it is difficult to draw robust conclusions about selection acting on the braincase. But in the following sections we expand upon the most significant correlations (or lack thereof) between shape and ecology/life history in the braincase and comment on potential functional/evolutionary relationships that they imply.

### Decoupling of the elasmobranch jaws and braincase reduces constraint and alters ecological signal

The most notable difference between our results and those recovered from other vertebrates is the absence of any significant relationship between neurocranium geometry and diet (Table 1). In other groups prey acquisition/handling is thought to be amongst the most significant selective pressures acting throughout the cranium, and as a result neurocranium shape frequently correlates with aspects of diet and trophic ecology^2,44,45^. In this regard the elasmobranch cranium displays mosaic evolution, where different parts of the skull show contrasting patterns of ecological signal. Whilst mandible shape does indeed correlate with some aspects of diet^46^, this signal is virtually absent from the neurocranium (Table 1). In most tetrapods there is an intimate musculoskeletal integration between the mandibles and cranium. In teleosts, the maxilla and premaxillar are highly kinetic and the upper jaw and suspensorium can be tightly connected to the braincase. By contrast the elasmobranch cranium is uniquely decoupled, with a few, often highly kinetic skeletal connections between the braincase and jaws^39,40,47–49^. Thus we might predict that elasmobranch neurocranial geometry may have a different degree of correlation with diet. However, we caution about generalising too strongly. There are very clear instances, such as in heterodontiform sharks, where major excursions of geometry associate with specialisation in feeding such as durophagy. A lack of clear signal between neurocranial shape and diet could be masked by some of the more extreme shape divergences such as those seen in hammerheads and pristiphoriform sharks.

Compared to many other vertebrate groups the elasmobranch neurocranium is highly integrated and composed of relatively few skeletal units separated by discrete sutures (Table 2; Supplementary Table S7)^3,50,51^. This suggests levels of correlation between the geometry of different regions of the neurocranium in elasmobranchs is amongst the highest observed in vertebrates. Some clades (such as pelagarian teleosts) display similar levels of integration, however in most vertebrate groups the cranium comprises at least five (and in many cases more than 10) discrete modules^3,50,51^. High levels of integration between subunits complex morphological structures are thought to be underlain by positive trait correlations, enhancing the response to selection and facilitating rapid, correlated morphological evolution^52–54^. The loss of skeletal connections between the braincase and the jaws may have released the neurocranium from significant constraint, enabling it to evolve under selective regimes less dominated by prey handling. Evolutionary constraint upon the braincase resulting from articulation with the jaws has been reported in other groups^55^. Indeed, many of the morphological ‘outliers’ amongst elasmobranchs, such as hammerhead sharks, display highly conserved jaw articulation despite exploration of other regions of neurocranial morphospace. Importantly, this does not necessitate those connections between the jaws and neurocranium were lost in elasmobranchs to directly facilitate increased braincase integration—this transition is also cited as having provided elasmobranchs with unparalleled behavioural/morphological flexibility with regards to the feeding apparatus^35^. It is clear however that the anatomical decoupling of the jaws and the braincase in elasmobranchs has enabled both structures to evolve independently, acquiring different patterns of ecological signal in the process.

### Relationships between braincase shape and other ecological variables

Herein correlations between neurocranium shape and ecology in Elasmobranchii are restricted to three broad categories (water depth, ‘water conditions’ and latitude), with most other correlations being lost when taking phylogeny into account (Tables 1,2; Supplementary Table S4). Whilst the absence of rate differences between modules in the selachian neurocranium (Supplementary Table S5; Supplementary Table S6) provides rudimentary evidence of natural selection across the braincase^56,57^, this does not match observed patterns of ecological signal, which appear to differ significantly between different regions of the braincase (Table 2). As mentioned previously, the orbit and the rostrum were found to be the most variable regions of the neurocranium, which is notable given the hypothesised functional importance of these structures (Table 2; Supplementary Table S7)^35–37^. In the following sections we expand on the three major correlations between braincase shape and ecology, commenting on their potential implications for form-function relationships and morphological evolution in elasmobranchs.

Anterior neurocranium shape in elasmobranchs is correlated with multiple measures of water depth (Table 2; Supplementary Table S7). Specifically, water depth is correlated with the relative size of the rostral cartilages and orbital processes compared to the rest of the braincase (Supplementary Figures S18-25). Taxa inhabiting deeper waters are characterised by large, robust orbital processes, expansion of the anterior braincase and the presence of substantial rostral cartilages, whereas shallow-water taxa are typified by smaller orbital processes and small or non-existent rostral cartilages (Fig. 4; Supplementary Figures S18–25). Interestingly when comparing between batoids and selachian some differences arise: in the former orbit morphology is not correlated with water depth and rostrum morphology correlates only with minimum water depth, whereas in selachian maximum water depth correlates with orbit morphology and minimum water depth is the only water depth measure not to correlate with rostrum shape (Table 2). These results are of interest given the high functional importance of both the orbit and the rostrum: the former houses the visual sensory system, whereas the rostrum is the anteriormost portion of the elasmobranch skeleton, and is key to hydrodynamic performance and the distribution of sensory structures such as the ampullae of Lorenzini^35–37^. In some marine vertebrates cranial morphology is known to vary with water depth due to its effects on pressure regime and the distribution/sensitivity of sensory structures^58–60^. Large orbits are often seen in deepwater taxa, where they are thought to improve visual performance or represent adaptations to pressure (e.g. scleral capsules). However, a paucity of functional studies comparing kinematics and sensory capabilities between shallow and deepwater elasmobranchs largely precludes further speculation. Nevertheless, there is some correlation between elasmobranch neurocranial morphology and water depth, suggesting that water depth may be an important driver of neurocranial shape variation, as has been found in other regions of the elasmobranch skeleton^46^.

Neurocranium morphology also appears to be influenced by a species’ position on the freshwater-marine continuum. In Selachii (but not Batoidea) all modules were correlated with water parameters (marine, brackish, freshwater)—with correlation being strongest in the occipital module (Table 2). Taxa capable of inhabiting brackish environments generally have dorsoventrally expanded and laterally compressed occipital regions compared to taxa that exclusively inhabit marine environments (Supplementary Figure S7). It is difficult to speculate as to what if any adaptive significance this shape variation may hold, as again the necessary functional studies have not been performed. Theoretically occiput shape could relate to the mechanical properties of the anterior vertebral column, but occiput shape is also likely to correlate strongly with the morphology of the internal semicircular canals. There are likely to be hydrodynamic and sensory differences between marine and brackish environments, but these are difficult to quantify through existing literature, and in any case disentangling how these differences might relate to occiput shape variation observed in this study is impossible at this time. We are also unable to speculate on why this correlation between ability to enter brackish environments and occiput morphology should be restricted to Selachii; this would be an intriguing avenue for further study. It is clear however that at least in the case of selachian elasmobranchs, ecological characteristics of environments differing in water chemistry may play some role in shaping neurocranial geometry.

Latitude correlates significantly with the shape of the posterior neurocranium in batoids and all modules in Selachii, however this result is difficult if not impossible to interpret on the basis of existing data (Table 2). Species inhabiting tropical latitudes are more likely to exhibit a broad ‘NE’ type neurocranium, with lateral compression and dorsoventral elongation of the occiput, orbit and rostrum associated with higher latitudes (Fig. 4; Supplementary Figure S10-16). Unlike water depth and water parameters, correlation between latitude and neurocranium shape is broadly similar across Elasmobranchii, with batoid rostrum morphology being the only module across either Batoidea or Selachii to lack this correlation (Table 2). Biogeography is a significant determinant of the composition of ecological communities^61^, potentially enacting various selective pressures upon neurocranial shape. Latitudinal gradients in morphology are known from several vertebrate clades, typically linked to latitudinally-mediated differences in diet or habitat usage^1,62^. Intriguingly in elasmobranchs, morphologies exhibited at high latitudes appear similar to those found in deepwater taxa and those in brackish environments (Fig. 4; Supplementary Figure S7; Supplementary Figures S10–25). This is indicative of either a monotonic response to temperature, or one-to-many mapping of form to function in the elasmobranch neurocranium. Our cluster analysis provides some evidence for one-to-many relationships, as within each morphotype exist taxa that differ substantially in terms of ecology (Fig. 4; Supplementary Table S1). Even in the case of the OE morphotype—where all taxa use modifications to the anterior cranium to assist in prey capture^37^ there is substantial variation in trophic/spatial ecology between taxa (Supplementary Table S1). Unfortunately, a lack of existing literature regarding functional morphology of neurocranium variation in elasmobranchs, the near ubiquity of latitude as a significant covariate of shape, and the rather coarse nature of species-specific ecological information makes inference of the nature of the relationship between neurocranial shape and latitude at best speculative. Nevertheless, it is clear that biogeography—or some correlate of biogeography- is an important factor in shaping elasmobranch neurocranial morphology.

### Batoids have more modular braincases than selachians

Whilst the neurocranium of both batoids and selachians displays global integration (Supplementary Table S7), there are marked differences between the two in terms of the number of independently evolving ‘modules’ present (Fig. 5). Selachii appear to possess three neurocranial modules, batoids possess four, and integration between modules is generally lower in the latter (Supplementary Table S7). In batoids the occipital and otic regions (which form a single module in Selachii) are separate, and the nasal capsules contribute to the rostrum module rather than the orbit module (Fig. 5; Supplementary Table S2). In Selachii the jaws are articulated to the braincase through the hyomandibular cartilages and a suspensory ligament joining the anterior neurocranium to the palatoquadrate^63,64^. In batoids this ethmopalatine ligament is lost^47,65^, meaning that the otic region of the neurocranium is more important to jaw articulation in batoids than in selachians. The loss of this ligamentous connection may thus have increased the functional separation of the otic and occipital regions of the batoid neurocranium. In this scenario, increased modularity between the otic and occipital regions is adaptive as it overcomes constraint imposed by subunits of a morphological structure that differ functionally. As a result, batoids have far greater motor control in the feeding apparatus and have evolved many innovations to the hyomandibular musculature to facilitate specialisation^5,47,65,66^ which may not have been possible in the absence of some degree of evolutionary independence between the occipital and otic regions. The loss of the ethmopalatine ligament would also likely have released the rostrum from evolutionary constraint, enabling greater optimisation of hydrodynamic function and the evolution of novel, batoid-specific roles in prey acquisition^37^. Of course, it is difficult to comment on the ancestral state of modularity in the elasmobranch braincase given these major differences between batoids and selachians. However, it does appear that the loss of the ethmopalatine ligament, combined with increased modularity in the posterior neurocranium has enabled batoids to evolve complex and flexible prey-acquisition strategies that may not otherwise have been possible.

### Correlates of evolutionary rate and morphological disparity

We recovered evidence of numerous correlations between aspects of elasmobranch life-history/ecology and both morphological disparity and rates of morphological evolution (Supplementary Table S5; Supplementary Table S6). Intriguingly these correlates of disparity and evolutionary rate differ from the correlates of shape itself (Tables 1,2; Supplementary Table S5; Supplementary Table S6). Morphological disparity was found to correlate with water parameters, much like some aspects of neurocranium shape (Table 2; Table S5), but also with factors such as reproductive mode and habitat (Supplementary Table S5). This is even more-so true in the case of evolutionary rate, which correlated significantly with almost all tested possible ecological and life-history factors (Supplementary Table S6). Given the large number of correlations present it is extremely difficult to extract any meaningful evolutionary inference from these results alone—although it may well be the case that at least some of these correlates to have a genuine influence over rates of evolution or morphological disparity. For example, rates of morphological evolution in the elasmobranch braincase appear to correlate inversely with latitude such that morphological evolution proceeds more rapidly in the tropics (Supplementary Table S6). This is in-line with the extensively studied latitudinal species diversity gradient, for which much support has been gathered from marine Osteichthyes^67,68^. These results should be taken with extreme caution however. Correlations between ecology and rates of evolution could equally result from the high dimensionality and low effective sample size of our data. There are no clear and interpretable trends present in our disparity results (Supplementary Table S5),but given that there are various ways of measuring morphological disparity^69^ future studies using different measures may uncover different results.

### Limitations and future work

There are several limitations to this study, predominantly relating to the range of data available. Future studies should—wherever possible—seek to increase taxonomic coverage, both extant and extinct. Although the preservation potential of the elasmobranch neurocranium is low^70^, preserved (fossilised) neurocrania are known and could be included in future studies. Only a single specimen for each species in this study, despite significant intraspecific variation in elasmobranch cranial morphology^71,72^ however we considered this approach valid as interspecific morphological differences sufficient to distinguish between species are thought to be present regardless of ontogenetic stage or sex^72^. More significant is that it is difficult to speculate on the selective regimes influencing the neurocranium, or the nature of putative trait correlations and modules, without a priori knowledge of the developmental/genetic basis of morphology^73^. Evo-devo studies (e.g.^74^) focussing on the neurocranium should form the major focus of future studies investigating elasmobranch braincase evolution. Finally, it is important to mention that all phylogenies, models of trait evolution, and calculations of ancestral states are intrinsically hypotheses, subject to revision upon the incorporation of additional data.

## Conclusion

The evolutionary history of the elasmobranch neurocranium is, much like the structure itself, complex and multifaceted. Nonetheless clear correlations between shape variation and ecology exist: water depth and biogeography (and possibly temperature by implication) appear to be important factors underlying neurocranium variation across Elasmobranchii. Despite global integration, there exist clear differences in ecological signal between different modules, particularly between the anterior and posterior neurocranium. Morphological evolution in the elasmobranch skull is intrinsically associated with innovation of the jaw suspension—facilitating increased evolutionary independence between the jaws and braincase. Not all of the results found in this study have clear evolutionary interpretation, but we hope that this will prompt further studies seeking to expand upon and explain these results. Specifically, we suggest that additional work is needed to discern form-function relationships in the elasmobranch neurocranium, the genetic/developmental basis of putative independent modules, and to better explain apparent correlations between ecology, rates of evolution and different measures of shape variation.

### Supplementary Information

Supplementary Information.

## Data Availability

All data and code generated in this project have been deposited in the following Figshare repository: 10.6084/m9.figshare.25388581.
