# Supplementary material for: Evolutionary trends in the elasmobranch neurocranium
Source: Sci Rep. 2024 May 20;14:11471. doi: 10.1038/s41598-024-62004-3 (PMC11106257; doi:10.1038/s41598-024-62004-3)
Supplement: Supplementary file 1 — Supplementary Information. [file 41598_2024_62004_MOESM1_ESM.pdf]

Supplementary Material for “Evolutionary trends in the elasmobranch neurocranium”

SupplementaryTable S1: full ecological dataset. Lat refers to latitude, repro refers to reproductive mode, trophic refers to trophic level. 'CTOL' refers to the Chondrichthyan Tree of Life website (Naylor, 2023). Abbreviations under status (conservation status) are as follows: LC (least concern), NT (near threatened), V (vulnerable), E (endangered), CE (critically endangered), DD (data deficient). Empty cells indicate that scanning information for a given specimen is unknown. Gridlines have been maintained to improve legibility, given the large size of this table. Cluster refers to neurocranium morphotype, where cluster 1 equates to the RE morphotype, cluster 2 to the OE morphotype, and cluster 3 to the NE morphotype.

species	clade1a	clade2	Data source	Scanner	Specimen	clade3	clade4	latitude	habitat	water	reproduction	trophic	lm	status	lat.median	lat.range	lat.min	lat.max	depth.min	depth.max	depth.median	depth.range	feeding	dental	cluster	Notes
Bathyraja_kincaidii	Batoidea	Rajiformes	CTOL	MUSC Force	USNM 103425	Arhynchobatidae	Bathyraja	temperate	bathydemersal	marine	oviparous			LC					119	1372	745.5	1253	suction-grasping	crushing	3	
Atlantoraja_platana	Batoidea	Rajiformes	CTOL	MUSC Force	USNM 55578	Arhynchobatidae	Atlantoraja	subtropical	demersal	marine	oviparous		72	E	30	20	20	40	19	181	100	162	suction-grasping	crushing	1	
Rioraja_agassizii	Batoidea	Rajiformes	CTOL			Arhynchobatidae	Rioraja	subtropical	demersal	marine	oviparous		42.5	V									suction-grasping	crushing	1	
Sympterygia_bonapartii	Batoidea	Rajiformes	CTOL	MUSC S64	USNM 55577	Arhynchobatidae	Sympterygia	temperate	demersal	marine	oviparous			NT	38	12	32	44					suction-grasping	crushing	3	
Pseudoraja_fischeri	Batoidea	Rajiformes	CTOL	MUSC Force	USNM_285422	Arhynchobatidae	Pseudoraja	tropical	pelagic	marine	oviparous			LC									suction-grasping	crushing	3	
Psammobatis_scobina	Batoidea	Rajiformes	CTOL	MUSC Force	USNM 228152	Arhynchobatidae	Psammobatis	subtropical	demersal	marine	oviparous			LC					40	450	245	410	suction-grasping	crushing	3	
Irolita_waitii	Batoidea	Rajiformes	CTOL	MUSC Force	CSIRO H 133-1	Arhynchobatidae	Irolita	temperate	demersal	marine	oviparous			LC	32.5	7	29	36	50	200	125	150	suction-grasping	crushing	3	
Insentiraja_subtilispinosa	Batoidea	Rajiformes	CTOL	AMNH HRCT	CSIRO H 2583-09	Arhynchobatidae	Insentiraja	tropical	bathydemersal	marine	oviparous			LC	26	10	21	31	970	1100	1035	130	suction-grasping	crushing	1	
Pavoraja_nitida	Batoidea	Rajiformes	CTOL	AMNH HRCT	CSIRO CA 2817	Arhynchobatidae	Pavoraja	temperate	demersal	marine	oviparous		31.9	LC	37	14	30	44	30	390	210	360	suction-grasping	crushing	3	
Brochiraja_leviveneta	Batoidea	Rajiformes	CTOL	AMNH HRCT	CSIRO H 3132-05	Arhynchobatidae	Brochiraja		bathydemersal	marine	oviparous			DD					300	1200	750	900	suction-grasping	crushing	1	
Okamejei_acutispina	Batoidea	Rajiformes	CTOL	AMNH HRCT	AMNH 258307 (cf.)	Rajidae	Okamejei	temperate	demersal	marine	oviparous			V					25	150	87.5	125	suction-grasping	crushing	1	
Malacoraja_senta	Batoidea	Rajiformes	CTOL	MUSC Force	USNM 386068	Rajidae	Malacoraja	temperate	bathydemersal	marine + brackish	oviparous			V	47	14	40	54	25	1436	730.5	1411	suction-grasping	crushing	1	
Amblyraja_radiata	Batoidea	Rajiformes	CTOL	MUSC Force	USNM 386063	Rajidae	Amblyraja	boreal	demersal	marine + brackish	oviparous		87.5	V	58.5	41	38	79	5	1540	772.5	1535	suction-grasping	crushing	1	
Leucoraja_erinacea	Batoidea	Rajiformes	CTOL	MUSC Force	USNM 386062	Rajidae	Leucoraja	temperate	demersal	marine	oviparous		37.5	LC	41	16	33	49	10	914	462	904	suction-grasping	crushing	3	
Gurgesiella_furvescens	Batoidea	Rajiformes	CTOL	AMNH HRCT	USNM 222258	Rajidae	Gurgesiella		bathydemersal	marine	oviparous			LC									suction-grasping	crushing	3	
Raja_eglanteria	Batoidea	Rajiformes	CTOL	MUSC S64	GMBL 02-155 (also GMBL 5557)	Rajidae	Raja	subtropical	demersal	marine + brackish	oviparous		53.5	LC	35	26	22	48	0	330	165	330	suction-grasping	crushing	1	
Hypnos_monopterygius	Batoidea	Torpediniformes	CTOL	MUSC S64	USNM 84374	Hypnidae	Hypnos	temperate	reef-associated	marine	ovoviviparous			LC	28	20	18	38	0	240	120	240			3	
Narcine_brasiliensis	Batoidea	Torpediniformes	CTOL	AMNH HRCT	AMNH 77069	Narcinidae	Narcine	subtropical	reef-associated	marine	ovoviviparous		28.8	NT	1	39	0	39	1	43	22	42			3	
Narcine_westraliensis	Batoidea	Torpediniformes	CTOL			Narcinidae	Narcine	subtropical	demersal	marine	unknown			LC	22.5	7	19	26	10	70	40	60			3	

Temera_hardwickii	Batoidea	Torpediniformes	CTOL	MUSC Force	Unasigned	Narkidae	Temera	tropical	demersal	marine	unknown			V	7.5	11	2	13							3	Missing landmark 57
Narke_japonica	Batoidea	Torpediniformes	CTOL	AMNH HRCT	AMNH 258312	Narkidae	Narke	subtropical	reef-associated	marine	ovoviviparous			V	28.5	17	20	37							3	Missing landmark 57
Discopyge_tschudii	Batoidea	Torpediniformes	CTOL	MUSC S64	USNM 53439	Narcinidae	Discopyge	subtropical	demersal	marine	unknown			LC	35.5	41	15	56	5	165	85	160			3	
Diplobatis_ommata	Batoidea	Torpediniformes	CTOL	MUSC Force	USNM 321476	Narcinidae	Diplobatis	tropical	reef-associated	marine	unknown			LC	13	28	0	28	3	94	48.5	91			3	Missing landmark 57
Torpedo_fuscomaculata	Batoidea	Torpediniformes	CTOL	MUSC Force	USNM 320677	Torpedinidae	Torpedo	tropical	reef-associated	marine + brackish	ovoviviparous			DD	18.5	33	2	35	0	439	219.5	439			3	
Aptychotrema_vincentiana	Batoidea	Rhinopristiformes	CTOL	MUSC S64	CSIRO MUW 101	Trygonorrhinidae	Aptychotrema	temperate	demersal	marine	unknown			LC	29.5	21	19	40						crushing	1	
Rhina_ancylostoma	Batoidea	Rhinopristiformes	CTOL	MUSC S64	LACM 38117-38	Rhinidae	Rhina	tropical	reef-associated	marine	ovoviviparous		164	CE	4	42	0	42	3	90	46.5	87		crushing	2	
Rhynchobatus_springeri	Batoidea	Rhinopristiformes	CTOL	MUSC S64	AMNH 258310 (cf.)	Rhinidae	Rhynchobatus	tropical	pelagic	marine + brackish	unknown			CE	5	20	0	20	16	37	26.5	21		crushing	1	
Pristis_clavata	Batoidea	Rhinopristiformes	CTOL	WHOI	CSIRO H 2504	Pristidae	Pristis	tropical	demersal	marine + brackish	ovoviviparous			CE	2.5	25	0	25	1	20	10.5	19		crushing	1	
Pseudobatos lentiginosus	Batoidea	Rhinopristiformes	CTOL	MUSC S64	GMBL 74-37	Rhinobatidae	Rhinobatos	subtropical	reef-associated	marine	ovoviviparous			V	27	18	18	36	0	30	15	30		crushing	1	
Urolophus_aurantiacus	Batoidea	Myliobatiformes	CTOL	AMNH HRCT	AMNH 258305	Urolophidae	Urolophus	temperate	demersal	marine	ovoviviparous			V									suction-crushing		3	Missing landmark 57
Urobatis_concentricus	Batoidea	Myliobatiformes	CTOL	MUSC Force	USNM 87539	Urotrygonidae	Urobatis	tropical	demersal	marine	unknown			LC	22	18	13	31	1	35	18	34	suction-crushing		3	Missing landmark 57
Urotrygon_chilensis	Batoidea	Myliobatiformes	CTOL	AMNH HRCT	AMNH 233905	Urotrygonidae	Urotrygon	subtropical	demersal	marine	ovoviviparous			NT					1	60	30.5	59	suction-crushing		3	Missing landmark 57
Urotrygon_rogersi	Batoidea	Myliobatiformes	CTOL			Urotrygonidae	Urotrygon	tropical	demersal	marine	unknown		11.8	NT	12.5	27	0	27	2	30	16	28	suction-crushing		3	Missing landmark 57
Potamotrygon_motoro	Batoidea	Myliobatiformes	CTOL	AMNH HRCT	AMNH 97428	Potamotrygonidae	Potamotrygon	tropical	benthopelagic	freshwater	unknown			DD									suction-crushing		3	Missing landmark 57
Dasyatis_americana	Batoidea	Myliobatiformes	CTOL	MUSC S64	GMBL 72-158	Dasyatidae	Dasyatis	subtropical	reef-associated	marine + brackish	ovoviviparous		81.3	NT	16	36	0	36	0	53	26.5	53	suction-crushing	crushing	3	Missing landmark 57
Dasyatis_zugei	Batoidea	Myliobatiformes	CTOL	AMNH HRCT	AMNH 258174	Dasyatidae	Dasyatis	tropical	demersal	marine + brackish	ovoviviparous		18.7	V									suction-crushing	crushing	3	Missing landmark 57
Rhinoptera_bonasus	Batoidea	Myliobatiformes	CTOL	MUSC S64	GMBL 73-7	Rhinopteridae	Rhinoptera	tropical	benthopelagic	marine + brackish	ovoviviparous		65.3	V	1	42	0	42	0	22	11	22	suction-crushing		3	Missing landmark 57
Mobula_munkiana	Batoidea	Myliobatiformes	CTOL	UC Irvine Medical Center	SIO 85-34	Mobulidae	Mobula	tropical	pelagic	marine	ovoviviparous			V	12.5	32	0	32	0	15	7.5	15	filter feeding	filter feeding	2	
Pliotrema_warreni	Selachii	Pristiophoriformes	CTOL	MUSC Force	USNM 353830	Pristiophoridae	Pilotrema	subtropical	demersal	marine	ovoviviparous	4.2		LC	29.5	15	22	37	10	915	462.5	905			1	
Pristiophorus_nudipinnis	Selachii	Pristiophoriformes	CTOL	WHOI	CSIRO	Pristiophoridae	Pristiophorus	temperate	demersal	marine	ovoviviparous			LC	36	10	31	41	31	165	98	134			1	
Squatina_japonica	Selachii	Squatiniformes	Kamminga et al. (2017)			Squatinidae	Squatina	subtropical	demersal	marine	ovoviviparous			CE	30	22	19	41						clutching	3	
Squatina_nebulosa	Selachii	Squatiniformes	CTOL	AMNH HRCT	AMNH 258172	Squatinidae	Squatina		demersal	marine	ovoviviparous			E	34.5	25	22	47	0	200	100	200		clutching	3	
Squatina_dumeril	Selachii	Squatiniformes	CTOL			Squatinidae	Squatina	subtropical	bathydemersal	marine	ovoviviparous		99.5	LC	26	34	9	43	1	1375	688	1374		clutching	3	
Squatina_africana	Selachii	Squatiniformes	Kamminga et al. (2017)			Squatinidae	Squatina	subtropical	demersal	marine	ovoviviparous	4.2	91.5	NT	18	28	4	32		494				clutching	3	
Squatina_squatina	Selachii	Squatiniformes	Kamminga et al. (2017)			Squatinidae	Squatina	temperate	demersal	marine + brackish	ovoviviparous	4	147.5	CE	42	42	21	63	5	150	77.5	145		clutching	3	
Squalus_cubensis	Selachii	Squaliformes	Kamminga et al. (2017)			Squalidae	Squalus	subtropical	demersal	marine	ovoviviparous	4.2		LC	10	56	0	56	60	400	230	340	cutting	cutting-clutching	3	

Squalus_megalops	Selachii	Squaliformes	Kamminga et al. (2017)			Squalidae	Squalus	subtropical	demersal	marine	ovoviviparous	4.2	57	LC	9	60	0	60	30	750	390	720	cutting	cutting-clutching	3	
Squalus_suckleyi	Selachii	Squaliformes	Kamminga et al. (2017)			Squalidae	Squalus	subtropical	benthopelagic	marine + brackish	ovoviviparous		94.4	LC	11	38	29	67	15	1244	629.5	1229	cutting	cutting-clutching	3	
Oxynotus_centrina	Selachii	Squaliformes	CTOL	MUSC Force	USNM 206065	Oxynotidae	Oxynotus		bathydemersal	marine	ovoviviparous			E	26.5	70	0	70	60	1309	684.5	1249	cutting	cutting-clutching	3	
Heterodontus_francisci	Selachii	Heterodontiformes	CTOL	AMNH HRCT	AMNH 96795	Heterodontidae	Heterodontus	subtropical	demersal	marine	oviparous			DD	11	37	0	37	0	152	76	152	suction-crushing	clutching-grinding	3	
Eucrossorhinus_dasypogon	Selachii	Orectolobiformes	Kamminga et al. (2017)			Orectolobidae	Eucrossorhinus		reef-associated	marine	ovoviviparous			LC	11	22	1	23	2	50	26	48	suction-crushing	clutching	3	
Sutorectus_tentaculatus	Selachii	Orectolobiformes				Orectolobidae	Sutorectus	temperate	reef-associated	marine	oviparous			LC	30.5	9	26	35	0	35	17.5	35	suction-crushing	clutching	3	
Orectolobus_maculatus	Selachii	Orectolobiformes	CTOL	MUSC S64	USNM 50725	Orectolobidae	Orectolobus	temperate	reef-associated	marine	oviparous			LC	30	20	20	40	0	248	124	248	suction-crushing	clutching	3	
Stegostoma_fasciatum	Selachii	Orectolobiformes	CTOL	MUSC S64	LACM 38125-2	Stegostomatidae	Stegostoma	tropical	reef-associated	marine + brackish	oviparous	3.1		E	5.5	41	0	41	0	90	45	90	suction-crushing	clutching	3	
Ginglymostoma_cirratum	Selachii	Orectolobiformes	CTOL	MUSC S64	USNM 127110	Ginglymostomatidae	Ginglymostoma	subtropical	reef-associated	marine + brackish	ovoviviparous	3.8	235	V	4.5	44	0	44	0	130	65	130	suction-crushing	clutching	3	
Nebrius_ferrugineus	Selachii	Orectolobiformes	CTOL	MUSC S64	USNM 206988	Ginglymostomatidae	Nebrius	tropical	reef-associated	marine	ovoviviparous	4.1		V	4.5	36	0	36	0	70	35	70	suction-crushing	clutching	3	
Hemiscyllium_trispeculare	Selachii	Orectolobiformes	Kamminga et al. (2017)			Hemiscylliidae	Hemiscyllium	tropical	reef-associated	marine	oviparous			LC	15	14	8	22	0	50	25	50	suction-crushing	clutching	3	
Hemiscyllium_ocellatum	Selachii	Orectolobiformes	CTOL	AMNH HRCT	AMNH 44128	Hemiscylliidae	Hemiscyllium	tropical	reef-associated	marine	oviparous	3.5		LC	17.5	33	1	34	0	50	25	50	suction-crushing	clutching	3	
Hemiscyllium_strahani	Selachii	Orectolobiformes	Kamminga et al. (2017)			Hemiscylliidae	Hemiscyllium	tropical	reef-associated	marine	oviparous			V	7.5	5	5	10	3	18	10.5	15	suction-crushing	clutching	3	
Chiloscyllium_hasseltii	Selachii	Orectolobiformes	Kamminga et al. (2017)			Hemiscylliidae	Chiloscyllium	tropical	reef-associated	marine	oviparous			E	16.5	13	10	23	0	6	12	12	suction-crushing	clutching	3	
Chiloscyllium_griseum	Selachii	Orectolobiformes	CTOL	MUSC S64	USNM 205266	Hemiscylliidae	Chiloscyllium	tropical	reef-associated	marine + brackish	oviparous	3.7		E	12	34	0	34	5	100	52.5	95	suction-crushing	clutching	3	
Chiloscyllium_plagiosum	Selachii	Orectolobiformes	CTOL	AMNH HRCT	AMNH 258165	Hemiscylliidae	Chiloscyllium	tropical	reef-associated	marine	oviparous		56.5	NT	12.5	35	0	35	0	50	25	50	suction-crushing	clutching	3	
Poroderma_pantherinum	Selachii	Carcharhiniformes	CTOL			Scyliorhinidae	Poroderma	subtropical	reef-associated	marine	oviparous			LC	32	8	28	36	0	256	128	256	gouging	clutching	3	
Poroderma_africanum	Selachii	Carcharhiniformes	CTOL	MUSC Force	USNM 221658	Scyliorhinidae	Poroderma	subtropical	demersal	marine	oviparous	3.6	68.5	LC	32	8	28	36	0	100	50	100	gouging	clutching	3	
Scyliorhinus_meadi	Selachii	Carcharhiniformes	CTOL	MUSC S64	GMBL 8312	Scyliorhinidae	Scyliorhinus		bathydemersal	marine	oviparous	3.6		LC	24	8	20	28	300	600	450	300	gouging	clutching	3	
Cephaloscyllium_sarawakensis	Selachii	Carcharhiniformes	CTOL			Scyliorhinidae	Cephaloscyllium	subtropical	benthopelagic	marine	unknown		37.5	CE		0			10	200	105	190	gouging	clutching	3	
Schroederichthys_bivius	Selachii	Carcharhiniformes	CTOL	MUSC Force	USNM 114726	Atelomycteridae	Schroederichthys	subtropical	demersal	marine	oviparous	3.8		LC	39.5	33	23	56	14	78	46	64	gouging	clutching	3	
Schroederichthys_chilensis	Selachii	Carcharhiniformes	CTOL	MUSC Force	USNM 221636	Atelomycteridae	Schroederichthys	subtropical	demersal	marine	oviparous	4		LC	30	42	9	51					gouging	clutching	3	
Aulohalaelurus_labiosus	Selachii	Carcharhiniformes	CTOL	MUSC Force	USNM 170494	Atelomycteridae	Schroederichthys	subtropical	demersal	marine	oviparous			LC	32	8	28	36					gouging	clutching	3	
Atelomycterus_fasciatus	Selachii	Carcharhiniformes	CTOL	AMNH HRCT	CSIRO CA 4516	Atelomycteridae	Schroederichthys	tropical	demersal	marine	oviparous			LC	15.5	11	10	21	27	122	74.5	95	gouging	clutching	3	
Atelomycterus_marmoratus	Selachii	Carcharhiniformes	CTOL	MUSC Force	USNM 263409	Atelomycteridae	Schroederichthys	tropical	reef-associated	marine	oviparous			NT		0							gouging	clutching	3	
Hemipristis_elongata	Selachii	Carcharhiniformes	CTOL	MUSC S64	LACM 37712-1	Hemigaleidae	Hemipristis	tropical	demersal	marine	viviparous	4.3		V	3.5	41	0	41	1	130	65.5	129	gouging	cutting-clutching	1	
Hemigaleus_microstoma	Selachii	Carcharhiniformes	CTOL	MUSC Force	USNM 320633	Hemigaleidae	Hemigaleus	tropical	demersal	marine	viviparous	4.2		V	1	30	0	30					gouging	cutting-clutching	3	

Eusphyra_blochii	Selachii	Carcharhiniformes	CTOL	MUSC Force	USNM 205342	Sphyrnidae	Eusphyra	tropical	benthopelagic	marine + brackish	viviparous	4.1		E	5.5	31	0	31					gouging	cutting-clutching	2	
Sphyrna_zygaena	Selachii	Carcharhiniformes	CTOL	MUSC Force	USNM 325631	Sphyrnidae	Sphyrna	subtropical	pelagic	marine + brackish	viviparous	4.2	250	V	2	59	0	59	0	200	100	200	gouging	cutting-clutching	2	
Sphyrna_lewini	Selachii	Carcharhiniformes	CTOL	MUSC Force	USNM 203101	Sphyrnidae	Sphyrna	tropical	pelagic	marine + brackish	viviparous	4.1	210.5	CE	5.5	46	0	46	0	1000	500	1000	gouging	cutting-clutching	2	
Sphyrna_corona	Selachii	Carcharhiniformes	CTOL	MUSC Force	USNM 206987	Sphyrnidae	Sphyrna	tropical	demersal	marine	viviparous			CE	6.5	32	0	32	0	100	50	100	gouging	cutting-clutching	2	
Sphyrna_tiburo	Selachii	Carcharhiniformes	CTOL	AMNH HRCT	Needs AMNH assignment	Sphyrnidae	Sphyrna	subtropical	reef-associated	marine + brackish	viviparous	3.2	85	E	4.5	45	0	45	10	80	45	70	gouging	cutting-clutching	2	
Sphyrna_tudes	Selachii	Carcharhiniformes	CTOL	MUSC Force	USNM 159197	Sphyrnidae	Sphyrna	subtropical	benthopelagic	marine	viviparous	3.6		CE	5.5	57	0	57					gouging	cutting-clutching	2	
Sphyrna_media	Selachii	Carcharhiniformes	CTOL	MUSC Force	USNM 205377	Sphyrnidae	Sphyrna	tropical	demersal	marine	viviparous			CE	0.5	35	0	35					gouging	cutting-clutching	2	
Isogomphodon_oxyrhynchus	Selachii	Carcharhiniformes	CTOL	MUSC Force	USNM 221337	Carcharhinidae	Isogomphodon	tropical	demersal	marine + brackish	viviparous		115	CE	4	20	0	20					gouging		1	
Carcharhinus_altimus	Selachii	Carcharhiniformes	CTOL	MUSC S64	GMBL 03074 (Catalog #05558)	Carcharhinidae	Carcharhinus	subtropical	reef-associated	marine	viviparous	4.3	230	NT	7.5	46	0	46	12	810	411	798	gouging	cutting-clutching	3	
Carcharhinus_plumbeus	Selachii	Carcharhiniformes	CTOL	MUSC Force	GMBL 79-60	Carcharhinidae	Carcharhinus	subtropical	benthopelagic	marine + brackish	viviparous	4.1	166.3	E	1	45	0	45	0	500	250	500	gouging	cutting-clutching	3	
Triaenodon_obesus	Selachii	Carcharhiniformes	CTOL	MUSC Force	USNM 216792	Carcharhinidae	Triaenodon	tropical	reef-associated	marine	viviparous	4.2	107	V	0.5	30	0	30	1	330	165.5	329	gouging	cutting	3	
Prionace_glauca	Selachii	Carcharhiniformes	Kamminga et al. (2017)			Carcharhinidae	Prionace	subtropical	pelagic	marine + brackish	viviparous	4.1	199.1	NT	8	71	0	71	1	1082	541.5	1081	gouging	cutting	1	
Carcharhinus_galapagensis	Selachii	Carcharhiniformes	CTOL	MUSC S64	GMBL Uncatalogued	Carcharhinidae	Carcharhinus	tropical	reef-associated	marine	viviparous	4.2	230	LC	1	36	0	36	1	286	143.5	285	gouging	cutting-clutching	3	
Negaprion_brevirostris	Selachii	Carcharhiniformes	CTOL	MUSC S64	GMBL TL 990	Carcharhinidae	Negaprion	subtropical	reef-associated	marine + brackish	viviparous	4.2		V	6	45	0	45	0	92	46	92	gouging	cutting	3	
Loxodon_macrorhinus	Selachii	Carcharhiniformes	CTOL	MUSC Force	USNM 201721	Carcharhinidae	Loxodon	tropical	demersal	marine	viviparous	3.9	85	NT	2	34	0	34	7	100	53.5	93	gouging	cutting	1	
Scoliodon_macrorhynchos	Selachii	Carcharhiniformes	CTOL	AMNH HRCT	AMNH 258155	Carcharhinidae	Scoliodon	tropical	demersal	marine	unknown		42.9	NT									gouging	cutting	1	
Rhizoprionodon_terraenovae	Selachii	Carcharhiniformes	CTOL	MUSC Force	GMBL uncatalogued	Carcharhinidae	Rhizoprionodon	subtropical	demersal	marine + brackish	viviparous	4	87.5	LC	9	43	0	43	0	280	140	280	gouging	cutting	1	
Galeorhinus_galeus	Selachii	Carcharhiniformes	Kamminga et al. (2017)			Triakidae	Galeorhinus	subtropical	benthopelagic	marine	ovoviviparous		144.1	CE	6	70	0	70	0	1100	550	1100	gouging	clutching	1	
Triakis_semifasciata	Selachii	Carcharhiniformes	CTOL	MUSC Force	USNM 201722	Triakidae	Triakis	subtropical	demersal	marine + brackish	ovoviviparous	3.7	109.8	LC	32	26	19	45	0	156	78	156	crushing	crushing	3	
Hemitriakis_leucoperiptera	Selachii	Carcharhiniformes	CTOL	AMNH HRCT	USNM 325700	Triakidae	Hemitriakis	tropical	demersal	marine	ovoviviparous			CE	12.5	15	5	20					crushing	crushing	3	
Mustelus_canis	Selachii	Carcharhiniformes	CTOL	MUSC Force	GMBL 74-21	Triakidae	Mustelus	subtropical	demersal	marine + brackish	viviparous	3.6	99.8	NT	1	44	0	44	0	800	400	800	crushing	crushing	1	
Mustelus_mosis	Selachii	Carcharhiniformes	CTOL			Triakidae	Mustelus	tropical	demersal	marine	viviparous		67.5	NT	18.5	23	7	30					crushing	crushing	1	
Mustelus_asterias	Selachii	Carcharhiniformes	Kamminga et al. (2017)			Triakidae	Mustelus	temperate	demersal	marine	ovoviviparous	3.7	81	NT	38.5	45	16	61	0	350	175	350	crushing	crushing	3	
Mustelus_higmani	Selachii	Carcharhiniformes	Kamminga et al. (2017)			Triakidae	Mustelus		demersal	marine + brackish	viviparous	3.6		E	12.5	36	0	36	1	900	450.5	899	crushing	crushing	3	
Mustelus_manazo	Selachii	Carcharhiniformes	CTOL	AMNH HRCT	AMNH 258162	Triakidae	Mustelus	tropical	demersal	marine	ovoviviparous	3.6	65	E	17.5	45	0	45	0	360	180	360	crushing	crushing	3	
Leptocharias_smithii	Selachii	Carcharhiniformes	CTOL	MUSC Force	USNM 202651	Leptochariidae	Leptocharias	tropical	demersal	marine + brackish	viviparous			V	0.5	23	0	23	10	75	42.5	65	gouging		3	
Eridacnis_radcliffei	Selachii	Carcharhiniformes	CTOL	AMNH HRCT	AMNH 258156	Proscylliidae	Eridacnis	tropical	bathydemersal	marine	ovoviviparous	4	15.5	LC	6	22	0	22	71	766	418.5	695	gouging	clutching	3	

Proscyllium_magnificum	Selachii	Carchariniformes	CTOL			Proscylliidae	Proscyllium	tropical	pelagic	marine	unknown			NT									gouging		3	
Proscyllium_habereri	Selachii	Carchariniformes	CTOL	AMNH HRCT	AMNH 258157	Proscylliidae	Proscyllium	tropical	demersal	marine	oviparous			V	15.5	39	0	39	50	100	75	50	gouging		3	
Ctenacis_fehlmanni	Selachii	Carchariniformes	CTOL			Proscylliidae	Ctenacis	tropical	demersal	marine	oviparous			LC	10	2	9	11	70	170	120	100	gouging		3	
Gollum_attenuatus	Selachii	Carchariniformes	CTOL			Pseudotriakidae	Gollum		bathydemersal	marine	ovoviviparous	4.1		LC		0			120	660	390	540	gouging	clutching	3	
Bythaelurus_clevai	Selachii	Carchariniformes	CTOL			Pentanchidae	Bythaelurus		bathydemersal	marine	unknown			DD		0			425	500	462.5	75	gouging	clutching	3	
Figaro_boardmani	Selachii	Carchariniformes	CTOL	MUSC S64	CSIRO GT (AT?) 6290	Pentanchidae	Figaro	temperate	bathydemersal	marine	unknown			LC	34	20	24	44	128	823	475.5	695	gouging	clutching	3	
Asymbolus_pallidus	Selachii	Carchariniformes	CTOL			Pentanchidae	Asymbolus		bathypelagic	marine	oviparous			LC		0			225	402	313.5	177	gouging	clutching	3	
Asymbolus_analis	Selachii	Carchariniformes	CTOL	AMNH HRCT	CSIRO H 3431-02	Pentanchidae	Asymbolus	temperate	reef-associated	marine	oviparous			LC	35	6	32	38	10	180	95	170	gouging	clutching	3	
Galeus_gracilis	Selachii	Carchariniformes	CTOL			Pentanchidae	Galeus	tropical	bathydemersal	marine	unknown			DD	19	18	10	28	290	470	380	180	gouging	clutching	1	
Galeus_melastomus	Selachii	Carchariniformes	Kamminga et al. (2017)			Pentanchidae	Galeus		demersal	marine	oviparous	3.7	64.7	LC	38.5	51	13	64	55	1873	964	1818	gouging	clutching	1	
Parmaturus_melanobranchus	Selachii	Carchariniformes	CTOL			Pentanchidae	Parmaturus		bathydemersal	marine	oviparous			LC	19.5	3	18	21	540	810	675	270	gouging	clutching	3	
Parmaturus_bigus	Selachii	Carchariniformes	CTOL			Pentanchidae	Parmaturus		bathydemersal	marine	unknown			DD									gouging	clutching	3	
Galeus_eastmani	Selachii	Carchariniformes	CTOL			Pentanchidae	Galeus		bathydemersal	marine	oviparous		36.5	LC		0							gouging	clutching	3	
Galeus_sauteri	Selachii	Carchariniformes	CTOL	AMNH HRCT	AMNH 258160	Pentanchidae	Galeus		demersal	marine	oviparous			LC	23	4	21	25	60	90	75	30	gouging	clutching	1	
Apristurus_laurussonii	Selachii	Carchariniformes	Kamminga et al. (2017)			Pentanchidae	Apristurus		bathydemersal	marine	oviparous			LC	39	56	11	67	560	1550	1055	990	gouging	clutching	1	
Apristurus_macrostomus	Selachii	Carchariniformes	CTOL	AMNH HRCT	AMNH 258158	Pentanchidae	Apristurus		bathydemersal	marine	oviparous			LC		0							gouging	clutching	1	
Apristurus_pinguis	Selachii	Carchariniformes	CTOL			Pentanchidae	Apristurus		bathypelagic	marine	oviparous			LC		0			200	1000	600	800	gouging	clutching	3	
Holohalaelurus_regani	Selachii	Carchariniformes	CTOL	MUSC S64	UF 46769	Pentanchidae	Holohalaelurus		bathydemersal	marine	oviparous	4.2		LC	20.5	33	4	37					gouging	clutching	3	
Haploblepharus_edwardsii	Selachii	Carchariniformes	CTOL	MUSC S64	USNM 353828	Pentanchidae	Haploblepharus	subtropical	demersal	marine	oviparous	3.8		E	32	6	29	35	0	130	65	130	gouging	clutching	3	
Halaelurus_maculosus	Selachii	Carchariniformes	CTOL			Pentanchidae	Halaelurus	tropical	demersal	marine	unknown			NT									gouging	clutching	3	
Halaelurus_buergeri	Selachii	Carchariniformes	CTOL	MUSC Force	USNM 263408	Pentanchidae	Halaelurus	subtropical	demersal	marine	oviparous			E	29.5	19	20	39	80	100	90	20	gouging	clutching	3	
Mitsukurina_owstoni	Selachii	Lamniformes	CTOL	MUSC S64	HUMZ 204610	Mitsukurinidae	Mitsukurina		bathydemersal	marine	ovoviviparous		293	LC	3.5	55	0	55	30	1300	665	1270	gouging	tearing	1	
Odontaspis_ferox	Selachii	Lamniformes	CTOL	Children's memorial S64	BPBM 9334	Odontaspididae	Odontaspis		benthopelagic	marine	ovoviviparous			V	0.5	50	0	50	10	2000	1005	1990	gouging	tearing	3	
Pseudocarcharias_kamoharai	Selachii	Lamniformes	CTOL	Toshiba	LACM 45857	Pseudocarchariidae	Pseudocarcharias	subtropical	pelagic	marine	ovoviviparous		91.6	LC	0	40	0	40	0	590	295	590	gouging	tearing	3	
Alopias_vulpinus	Selachii	Lamniformes	Kamminga et al. (2017)			Alopidae	Alopias	subtropical	pelagic	marine	ovoviviparous	4.2	351	V	8	74	0	74	0	650	325	650	gouging	tearing	3	
Carcharias_taurus	Selachii	Lamniformes	CTOL	MUSC S64	UF 103326	Carchariidae	Carcharias	subtropical	reef-associated	marine	ovoviviparous	4.4	225	CE	1.5	48	0	48	1	191	96	190	gouging	tearing	3	
Lamna_ditropis	Selachii	Lamniformes	CTOL	Children's memorial S64	FMNH 117475	Lamnidae	Lamna	boreal	pelagic	marine	ovoviviparous			LC	44	44	22	66	0	650	325	650	gouging	tearing	1	

Lamna_nasus	Selachii	Lamniformes	Kamminga et al. (2017)			Lamnidae	Lamna	boreal	pelagic	marine	ovoviviparous	4.2	186.5	CE	8.5	76	0	76	0	1360	680	1360	gouging	tearing	1	
Carcharodon_carcharias	Selachii	Lamniformes	CTOL	WHOI	MCZ 171013	Lamnidae	Carcharodon	subtropical	pelagic	marine + brackish	ovoviviparous	4.5	475	V	1.5	61	0	61	0	1200	600	1200	gouging	cutting	3	
Isurus_oxyrinchus	Selachii	Lamniformes	CTOL	MUSC Force	GMBL 8446	Lamnidae	Isurus	subtropical	pelagic	marine	ovoviviparous	4.3		E	2.5	61	0	61	0	750	375	750	gouging	tearing	1	

SupplementaryTable S2: anatomical definitions for fixed and semilandmarks. Left and right identity assumes the neurocranium is being viewed from above.

Landmark number	Description
1	Foramen magnum (upper medial)
2	Foramen magnum (lower medial)
3	Foramen magnum (left medial)
4	Foramen magnum (right medial)
5	Foramen magnum semilandmark
6	Foramen magnum semilandmark
7	Foramen magnum semilandmark
8	Foramen magnum semilandmark
9	Foramen magnum semilandmark
10	Foramen magnum semilandmark
11	Foramen magnum semilandmark
12	Foramen magnum semilandmark
13	Occipital condyle (left upper)
14	Occipital condyle (left lower)
15	Occipital condyle (left lateral)
16	Occipital condyle (right upper)
17	Occipital condyle (right lower)
18	Occipital condyle (right lateral)
19	Left vagus foramen (upper medial)
20	Left vagus foramen (lower medial)
21	Left vagus foramen (left medial)

22	Left vagus foramen (right medial)
23	Right vagus foramen (upper medial)
24	Right vagus foramen (lower medial)
25	Right vagus foramen (left medial)
26	Right vagus foramen (right medial)
27	Left posteriomost ventral cartilage
28	Right posteriormost ventral cartilage
29	Semicircular canal (left)
30	Semicircular canal (right)
31	Endolymphatic foramen (left)
32	Endolymphatic foramen (right)
33	Preorbital process (left)
34	Postorbital process (left)
35	Preorbital process (right)
36	Postorbital process (right)
37	Left orbit semilandmark
38	Left orbit semilandmark
39	Left orbit semilandmark
40	Left orbit semilandmark
41	Left orbit semilandmark
42	Left orbit semilandmark
43	Right orbit semilandmark
44	Right orbit semilandmark
45	Right orbit semilandmark
46	Right orbit semilandmark
47	Right orbit semilandmark
48	Right orbit semilandmark
49	Left optic nerve foramen (upper medial)
50	Left optic nerve foramen (lower medial)
51	Left optic nerve foramen (left medial)
52	Left optic nerve foramen (right medial)
53	Right optic nerve foramen (upper medial)

54	Right optic nerve foramen (lower medial)
55	Right optic nerve foramen (left medial)
56	Right optic nerve foramen (right medial)
57	Anterior fontanelle (posterior)
58	Anterior fontanelle (anterior)
59	Anterior fontanelle semilandmark (left)
60	Anterior fontanelle semilandmark (left)
61	Anterior fontanelle semilandmark (left)
62	Anterior fontanelle semilandmark (left)
63	Anterior fontanelle semilandmark (right)
64	Anterior fontanelle semilandmark (right)
65	Anterior fontanelle semilandmark (right)
66	Anterior fontanelle semilandmark (right)
67	Right nasal capsule (upper medial)
68	Right nasal capsule (lower medial)
69	Right nasal capsule (rightmedial)
70	Right nasal capsule (left medial)
71	Left nasal capsule (upper medial)
72	Left nasal capsule (lowermedial)
73	Left nasal capsule (rightmedial)
74	Left nasal capsule (left medial)
75	Rostral cartilage (left anterior)
76	Rostral cartilage (central anterior)
77	Rostral cartilage (right anterior)
78	Rostral cartilage (left posterior)
79	Rostral cartilage (right posterior)

Supplementary Table S3: landmark configurations corresponding to hypotheses of modularity, modified from López-Romero et al. (2020)

Modularity hypothesis	Landmark configurations
2	Occipital: 1-28<br>Rostrum: 29-79
3a	Occipital: 1-32<br>Orbit: 33-56, 67-74<br>Rostrum: 57-66, 75-79
3b	Occipital: 1-32<br>Orbit: 33-56<br>Rostrum: 57-79
4a	Occipital: 1-32<br>Orbit: 33-56<br>Nasal capsules: 67-74<br>Rostrum: 57-66, 75-79
4b	Occipital: 1-26<br>Otic: 27-32<br>Orbit: 33-56<br>Rostrum: 57-79
5	Occipital: 1-26<br>Otic: 27-32<br>Orbit: 33-56<br>Nasal capsules: 67-74

Rostrum: 57-66, 75-79

Supplementary Table S4: model output for non-phylogenetic ANOVA / linear models including ecological variables as covariates of neurocranium shape

Covariate	SS	MS	R squared	F	Z	P	Df
Superorder	0.6467	0.64668	0.06436	8.8047	3.8307	1e-04	1,128
Order	4.0585	0.40585	0.40391	8.0634	5.7062	1e-04	10,119
Habitat	1.1301	0.226025	0.11247	3.1428	3.5922	2e-04	5,124
Latitude	0.3156	0.105183	0.0351	1.273	0.86585	0.1947	3,105
Water conditions	0.2987	0.149335	0.02972	1.9453	1.5226	0.0684	2,127
Status	0.8484	0.169687	0.08444	2.2872	3.0464	0.0012	5,124
Reproduction	1.3343	0.66713	0.14811	9.5622	5.6896	1e-04	2,110
Feeding type	1.6602	0.33204	0.23373	6.3444	3.7012	1e-04	5,104
Dental type	2.3498	0.39164	0.29762	7.2034	3.9332	1e-04	6,102
Body size	0.22499	0.224991	0.0725	3.5175	2.4425	0.0087	1,45
Trophic level	0.0921	0.092136	0.02694	1.1073	0.49894	0.3178	1,40
Minimum latitude	0.4293	0.42925	0.04822	5.3191	3.057	5e-04	1,105
Median latitude	0.5106	0.51059	0.05735	6.3884	3.4027	1e-04	1,105
Maximum latitude	0.2016	0.201580	0.02264	2.4325	1.8269	0.0326	1,105
Latitude range	0.5082	0.50818	0.05708	6.3564	3.229	1e-04	1,105
Minimum depth	0.1074	0.10736	0.0139	1.3954	0.92688	0.178	1,99
Median depth	0.1636	0.163559	0.02117	2.1416	1.6588	0.0509	1,99
Maximum depth	0.1787	0.178660	0.02313	2.344	1.8047	0.0343	1,99
Depth range	0.1865	0.186457	0.02414	2.4488	1.8683	0.0293	1,99

Supplementary Table S5: procrustes variances (disparity) for all covariate groups, with significant relationships (p<0.05) recovered from subsequent pairwise analyses

Covariate	Procrustes variance (disparity)	Significant relationships
Superorder	Batoidea: 8.00e-02<br>Selachii: 7.65e-02	N/A
Order	Carcharhiniformes: 6.48e-02<br>Heterodontiformes: 2.08e-02<br>Lamniformes: 4.35e-02<br>Myliobatiformes: 4.95e-02<br>Orectolobiformes: 3.81e-02<br>Pristiophoriformes: 2.92e-02<br>Rajiformes: 3.01e-02<br>Rhinopristiformes: 8.62e-02<br>Squaliformes: 1.91e-02<br>Squatiniformes: 1.74e-02<br>Torpediniformes: 3.47e-02	N/A
Habitat	Bathydemersal: 5.24e-02<br>Bathypelagic: 2.89e-02<br>Benthopelagic: 0.13<br>Demersal: 7.34e-02<br>Pelagic: 0.12<br>Reef-associated: 7.50e-02	Bathydemersal: Benthopelagic (p=0.025)<br>Bathydemersal: Pelagic (0.034)
Latitude	Boreal: 5.05e-02<br>Subtropical: 8.15e-02<br>Temperate: 7.73e-02<br>Tropical: 0.10	N/A
Status	Critically endangered: 0.14<br>Data deficient: 5.96e-02<br>Endangered: 0.10<br>Least concern: 6.64e-02<br>Near threatened: 5.48e-02	Critically endangered: Data deficient (p=0.04)<br>Critically endangered: Least concern (p=0.005)

	Vulnerable: 7.73e-02	Critically endangered: Near threatened (p=0.007)<br>Critically endangered: Vulnerable (p=0.021)
Water parameters	Freshwater: 3.80e-02<br>Marine: 7.20e-02<br>Marine + brackish: 0.13	N/A
Reproductive mode	Oviparous: 4.46e-02<br>Ovoviviparous: 9.95e-02<br>Viviparous: 0.12	Oviparous: Ovoviviparous (p=0.001)<br>Oviparous: Viviparous (p=0.002)
Feeding type	Crushing: 3.01e-02<br>Cutting: 1.91e-02<br>Filter feeding: 2.39e-02<br>Gouging: 6.77e-02<br>Suction crushing: 4.68e-02<br>Suction grasping: 3.14e-02	N/A
Dental type	Clutching: 4.54e-02<br>Clutching grinding: 2.25e-03<br>Crushing: 6.26e-02<br>Cutting: 4.17e-02<br>Cutting clutching: 0.11<br>Filter feeding: 1.07e-02<br>Tearing: 6.45e-02	Cutting: Clutching: Clutching (p=0.001)<br>Cutting clutching: Clutching grinding (p=0.046)<br>Cutting clutching: Crushing (p=0.009)<br>Cutting clutching: Cutting (p=0.014)

Supplementary Table S6: Evolutionary rates for all covariate groups, with significant rate ratios indicated in bold

Covariate	Evolutionary rate	Rate ratio	P value
Superorder	Batoidea: 2.35e-06<br>Selachii: 4.22e-06	**1.7962**	**0.001**
Order	Carcharhiniformes: 2.51e-06<br>Heterodontiformes: 1.41e-06<br>Lamniformes: 1.90e-06<br>Myliobatiformes: 2.45e-06<br>Orectolobiformes: 1.64e-05<br>Pristiophoriformes: 5.34e-06<br>Rajiformes: 2.02e-06<br>Rhinopristiformes: 4.06e-06<br>Squaliformes: 1.40e-06<br>Squatiniformes: 1.82e-06<br>Torpediniformes: 1.83e-06	**11.7193**	**0.001**
Habitat	Bathydemersal: 1.86e-06<br>Bathypelagic: 1.26e-06<br>Benthopelagic: 4.16e-06<br>Demersal: 1.99e-06<br>Pelagic: 4.03e-06<br>Reef-associated: 8.09e-06	**6.4202**	**0.001**
Latitude	Boreal: 1.73e-06<br>Subtropical: 2.06e-06<br>Temperate: 1.73e-06<br>Tropical: 6.76e-06	**3.9032**	**0.001**
Status	Critically endangered: 4.31e-06	**10.0038**	**0.001**

	Data deficient: 1.62e-06<br>Endangered: 1.62e-05<br>Least concern: 1.79e-06<br>Near threatened: 2.05e-06<br>Vulnerable: 1.86e-06		
Water parameters	Freshwater: 7.19e-07<br>Marine: 2.87e-06<br>Marine + brackish: 6.68e-06	**9.2964**	**0.001**
Reproductive mode	Oviparous: 5.35e-06<br>Ovoviviparous: 2.28e-06<br>Viviparous: 4.26e-06	**2.3418**	**0.001**
Feeding type	Crushing: 2.34e-06<br>Cutting: 1.02e-06<br>Filter feeding: 6.96e-06<br>Gouging: 2.44e-06<br>Suction crushing: 1.00e-05<br>Suction grasping: 2.06e-06	**9.8549**	**0.001**
Dental type	Clutching: 5.30e-06<br>Clutching grinding: 1.30e-06<br>Crushing: 2.36e-06<br>Cutting: 2.15e-06<br>Cutting clutching: 4.66e-06<br>Filter feeding: 6.26e-06<br>Tearing: 1.97e-06	**4.8374**	**0.007**

Supplementary Table S7: full integration results for the elasmobranch, selachimorph and batoid datasets. Statistically significant integration between modules ($p<0.05$) is denoted by bold typeface.

Group	Module-module integration	Global integration
Elasmobranchii	**Occipital-orbit: 0.9483**<br>**Occipital-rostrum: 0.6977**<br>**Rostrum-orbit: 0.8016**	**-1.430914**
Batoidea	**Occipital-orbit: 0.8366**<br>**Occipital-otic: 0.7782**<br>**Occipital-rostrum: 0.7714**<br>**Orbit-otic: 0.6189**<br>**Orbit-rostrum: 0.7563**<br>**Rostrum-otic: 0.5656**	**-1.455339**
Selachimorpha	**Occipital-orbit: 0.9511**<br>**Occipital-rostrum: 0.7848**<br>**Rostrum-orbit: 0.8108**	**-1.401445**

Supplementary Table S8: module-based PMANOVA and PGLS analyses. Significant results (p<0.05) are presented in bold.

Dataset	Module	Covariate	Z	P
**Elasmobranchii**	**Occipital**	**Water parameters**	**2.87**	**0.01**
		**Latitude (minimum)**	**3.22**	**<0.01**
		**Latitude (median)**	**2.52**	**<0.01**
		**Latitude (maximum)**	**2.70**	**0.01**
		**Latitude (range)**	**3.16**	**0.01**
		**Depth (maximum)**	**1.92**	**0.02**
		**Depth (range)**	**1.78**	**0.03**
		Superorder	-2.59	0.98
		Order	-2.00	1.00
		Dental type	-1.12	0.89
		Depth (median)	1.63	0.06
		Depth (minimum)	0.70	0.21
		Feeding type	-1.72	0.98
		Habitat	-0.39	0.59
		Latitude (qualitative)	-0.95	0.80
		Body size	0.50	0.31
		Reproductive mode	-0.51	0.66
		Status	-0.87	0.76
		Trophic level	0.95	0.18
	**Orbit**	**Water parameters**	**2.52**	**0.01**
		**Body size**	**1.90**	**0.03**
		**Latitude (minimum)**	**2.66**	**<0.01**
		**Latitude (median)**	**2.86**	**<0.01**
		**Latitude (maximum)**	**2.83**	**<0.01**
		**Latitude (range)**	**2.64**	**<0.01**
		**Depth (median)**	**2.39**	**0.04**
		**Depth (maximum)**	**2.39**	**0.01**
		**Depth (range)**	**2.19**	**0.01**
		Superorder	-2.10	0.98
		Order	-1.24	0.90
		Dental type	0.93	0.17
		Depth (minimum)	0.06	0.47
		Feeding type	0.33	0.37

		Habitat	1.45	0.07
		Latitude (qualitative)	-0.42	0.67
		Reproductive mode	1.51	0.06
		Status	0.15	0.44
		Trophic level	1.17	0.12
		**Water parameters**	**2.86**	**0.01**
		**Latitude (minimum)**	**3.10**	**<0.01**
		**Latitude (median)**	**2.74**	**<0.01**
		**Latitude (maximum)**	**2.86**	**<0.01**
		**Latitude (range)**	**3.03**	**<0.01**
		**Depth (median)**	**2.03**	**0.02**
		**Depth (maximum)**	**2.10**	**0.01**
		**Depth (range)**	**1.76**	**0.04**
		Superorder	-1.90	0.97
	**Rostrum**	Order	1.70	0.06
		Dental type	-0.10	0.55
		Depth (minimum)	1.43	0.07
		Feeding type	-0.38	0.66
		Habitat	0.56	0.28
		Latitude (qualitative)	0.85	0.20
		Body size	0.26	0.40
		Reproductive mode	0.98	0.17
		Status	-1.39	0.92
		Trophic level	0.90	0.19
		**Latitude (qualitative)**	**1.98**	**0.02**
		**Latitude (minimum)**	**1.67**	**0.05**
		Order	0.14	0.44
		Dental type	0.40	0.37
		Depth (maximum)	-0.00	0.50
		Depth (median)	0.30	0.39
		Depth (minimum)	0.50	0.31
		Depth (range)	-0.48	0.67
**Batoidea**	**Occipital**	Feeding type	0.14	0.44
		Habitat	-0.99	0.83
		Latitude (maximum)	-0.26	0.58
		Latitude (median)	1.22	0.67
		Latitude (range)	1.27	0.80
		Body size	-0.66	0.72
		Reproductive mode	-1.13	0.87
		Status	0.33	0.37
		Water parameters	0.16	0.43
	**Otic**	**Habitat**	**1.84**	**0.03**

	**Latitude (minimum)**	**2.07**	**0.02**
	**Latitude (median)**	**2.21**	**0.01**
	**Latitude (maximum)**	**2.06**	**0.02**
	Order	-1.79	0.96
	Dental type	1.00	0.20
	Depth (maximum)	-0.53	0.70
	Depth (median)	0.00	0.49
	Depth (minimum)	0.53	0.29
	Depth (range)	-1.01	0.84
	Feeding type	0.57	0.32
	Latitude (qualitative)	0.42	0.34
	Latitude (range)	0.49	0.31
	Body size	0.78	0.23
	Reproductive mode	-1.97	0.98
	Status	-0.62	0.73
	Water parameters	0.78	0.24
**Orbit**	**Dental type**	**1.87**	**0.04**
	**Body size**	**2.37**	**<0.01**
	Order	-0.63	0.73
	Depth (maximum)	0.36	0.36
	Depth (median)	0.78	0.22
	Depth (minimum)	0.62	0.27
	Depth (range)	-0.80	0.79
	Feeding type	1.73	0.05
	Habitat	0.40	0.34
	Latitude (qualitative)	-0.01	0.50
	Latitude (maximum)	0.23	0.42
	Latitude (median)	-1.43	0.92
	Latitude (minimum)	-1.51	0.93
	Latitude (range)	0.61	0.28
	Reproductive mode	-2.95	1.00
	Status	-0.04	0.51
	Water parameters	0.25	0.39
**Rostrum**	**Depth (minimum)**	**1.66**	**0.05**
	Order	1.61	0.05
	Dental type	0.95	0.16
	Depth (maximum)	-0.38	0.64
	Depth (median)	0.76	0.23
	Depth (range)	-1.62	0.95
	Feeding type	0.70	0.26
	Habitat	-0.03	0.51
	Latitude (qualitative)	0.32	0.37
	Latitude (maximum)	0.33	0.37

		Latitude (median)	0.11	0.45
		Latitude (minimum)	0.27	0.39
		Latitude (range)	-0.17	0.56
		Body size	0.70	0.25
		Reproductive mode	-2.31	0.99
		Status	0.53	0.30
		Water parameters	-1.10	0.86
	Occipital	**Water parameters**	**3.20**	**<0.01**
		**Trophic level**	**2.21**	**0.01**
		**Latitude (minimum)**	**3.05**	**0.01**
		**Latitude (median)**	**2.43**	**0.01**
		**Latitude (maximum)**	**2.72**	**0.01**
		**Latitude (range)**	**3.22**	**0.01**
		**Depth (maximum)**	**1.91**	**0.02**
		**Depth (range)**	**1.78**	**0.03**
		Order	-0.67	0.73
		Habitat	-0.29	0.55
		Latitude (qualitative)	-1.14	0.87
		Status	-0.92	0.78
		Dental type	-1.19	0.90
		Depth (median)	1.64	0.06
		Depth (minimum)	0.74	0.20
		Feeding type	-0.30	0.56
		Body size	0.50	0.31
Selachii		Reproductive mode	-0.80	0.77
	Orbit	**Water parameters**	**2.67**	**<0.01**
		**Latitude (minimum)**	**2.54**	**0.01**
		**Latitude (median)**	**2.61**	**<0.01**
		**Latitude (maximum)**	**2.64**	**0.01**
		**Latitude (range)**	**2.52**	**0.01**
		**Depth (maximum)**	**2.23**	**0.01**
		**Depth (range)**	**2.10**	**0.02**
		Order	-0.93	0.83
		Dental type	0.34	0.37
		Depth (median)	1.63	0.05
		Depth (minimum)	-0.64	0.73
		Feeding type	-0.65	0.74
		Habitat	1.22	0.11
		Latitude (qualitative)	-0.42	0.66
		Body size	1.57	0.06
		Reproductive mode	1.28	0.10
		Status	-0.31	0.62
		Trophic level	1.23	0.11

**Rostrum**	**Water parameters**	**3.19**	**0.01**
	**Latitude (minimum)**	**3.03**	**<0.01**
	**Latitude (median)**	**2.73**	**<0.01**
	**Latitude (maximum)**	**3.00**	**<0.01**
	**Latitude (range)**	**3.02**	**<0.01**
	**Depth (median)**	**1.82**	**0.03**
	**Depth (maximum)**	**2.29**	**0.01**
	**Depth (range)**	**2.31**	**0.01**
	Order	1.64	0.07
	Dental type	-0.48	0.69
	Depth (minimum)	0.26	0.39
	Feeding type	-2.38	0.99
	Habitat	0.57	0.27
	Latitude (qualitative)	0.37	0.35
	Body size	0.00	0.50
	Reproductive mode	1.17	0.12
	Status	-0.37	0.65
	Trophic level	1.51	0.07

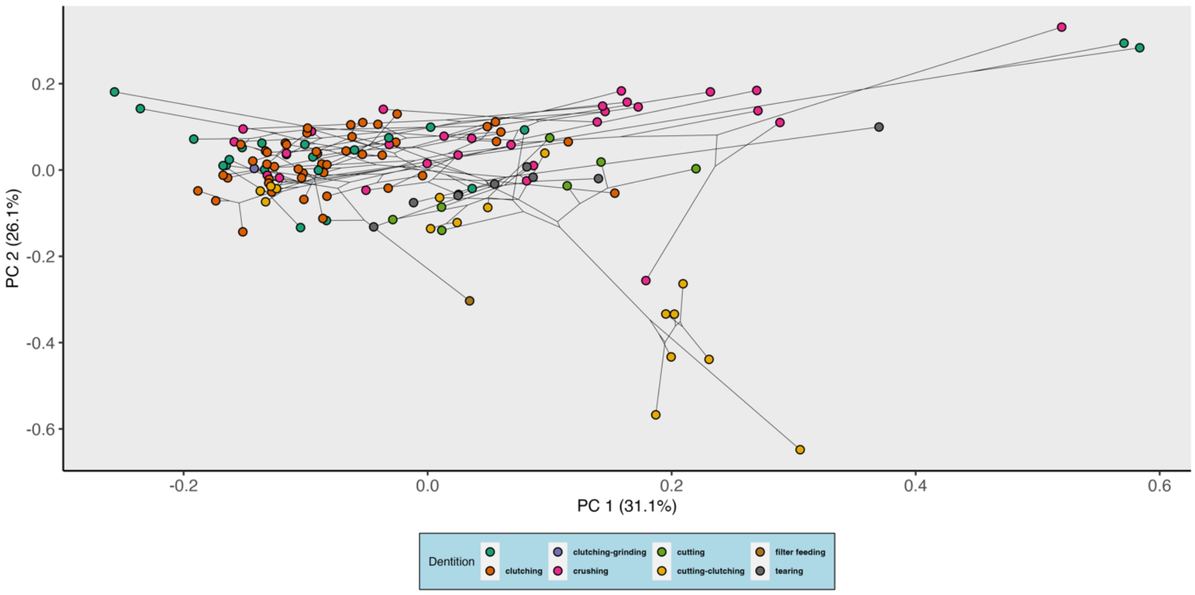

Supplementary Figure S1: phylomorphospace reconstruction demonstrating qualitative relationships (or lack thereof) between neurocranium geometry and dental type.

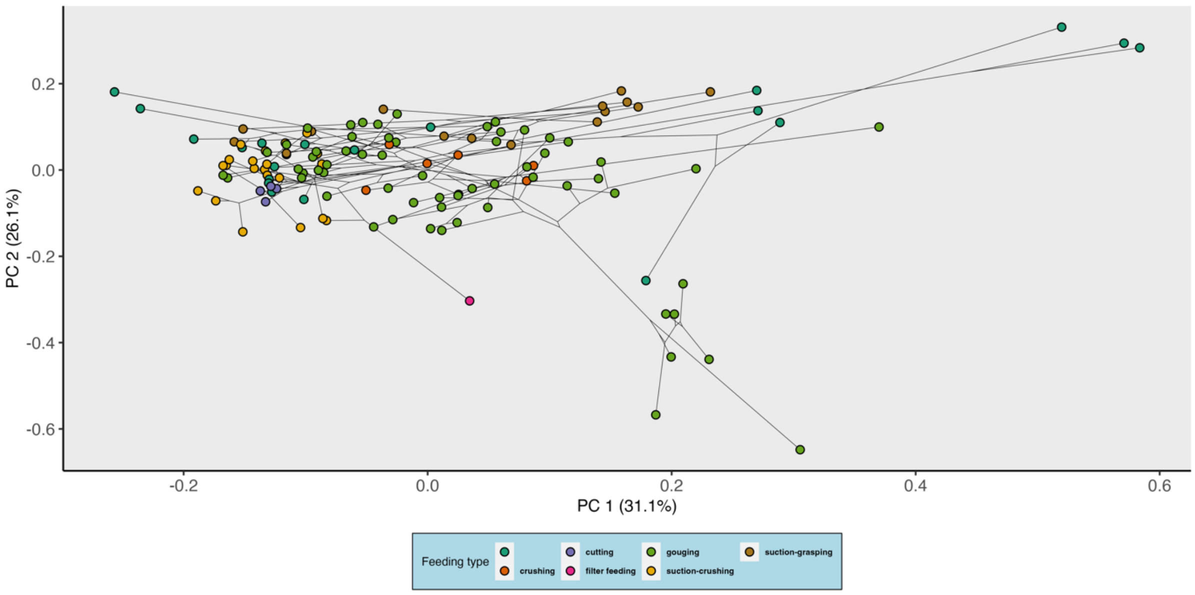

Supplementary Figure S2: phylomorphospace reconstruction demonstrating qualitative relationships (or lack thereof) between neurocranium geometry and feeding type.

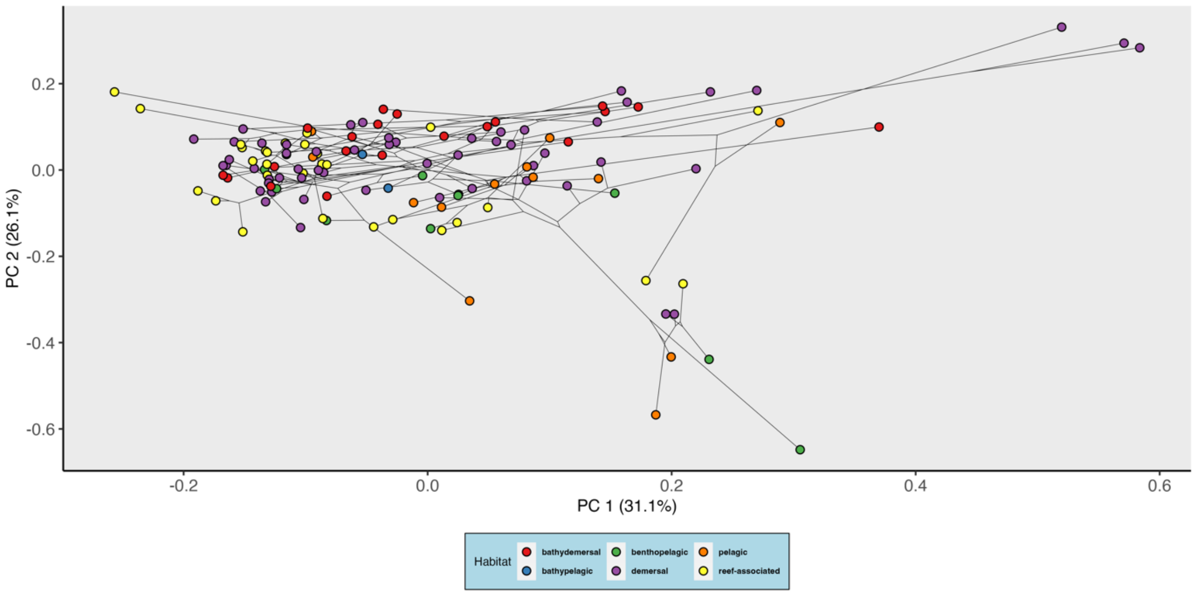

Supplementary Figure S3: phylomorphospace reconstruction demonstrating qualitative relationships (or lack thereof) between neurocranium geometry and habitat.

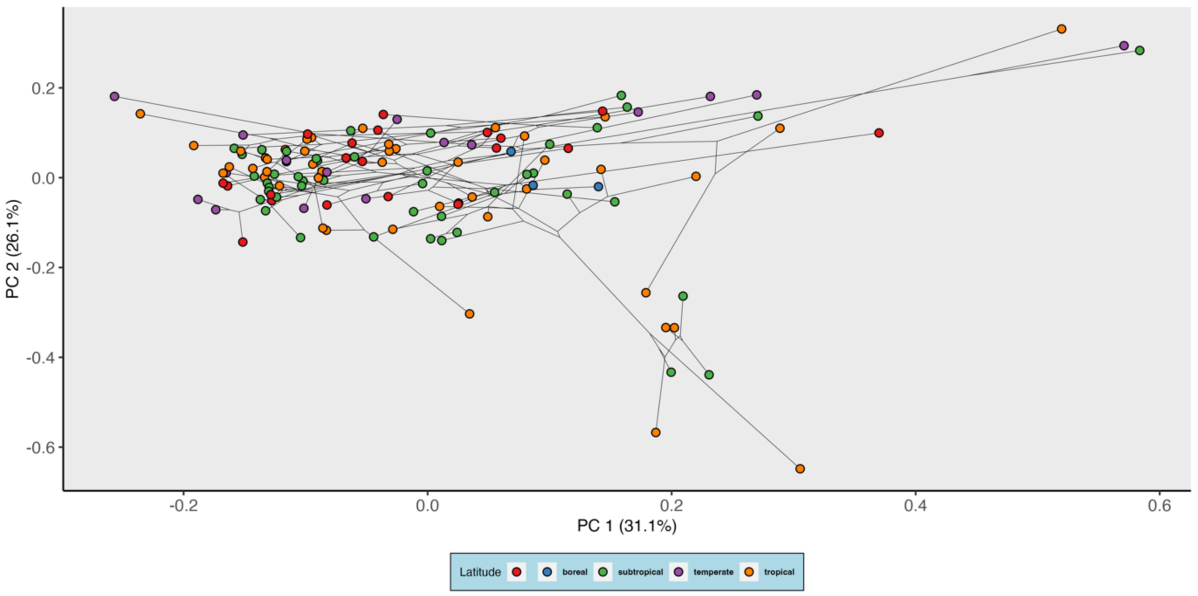

Supplementary Figure S4: phylomorphospace reconstruction demonstrating qualitative relationships (or lack thereof) between neurocranium geometry and latitude.

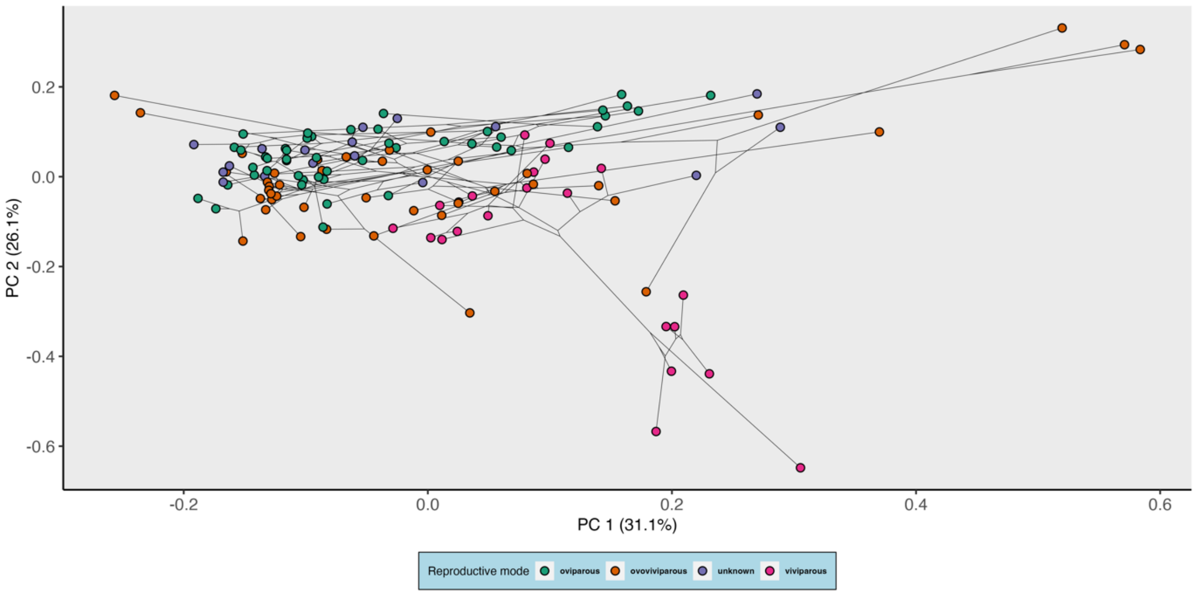

Supplementary Figure S5: phylomorphospace reconstruction demonstrating qualitative relationships (or lack thereof) between neurocranium geometry and reproductive mode.

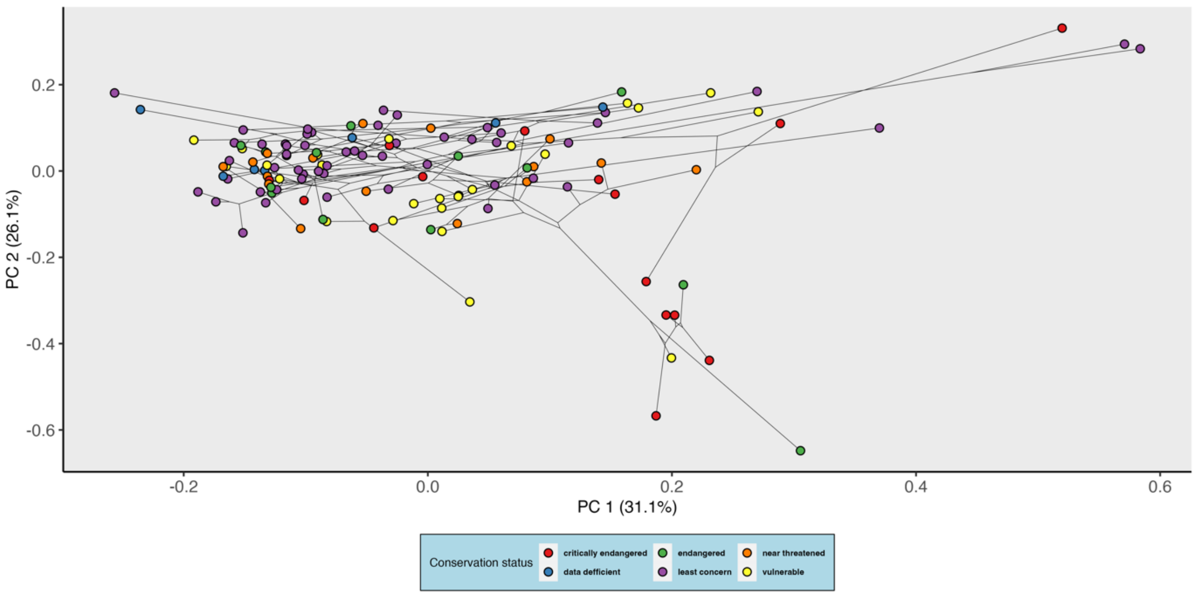

Supplementary Figure S6: phylomorphospace reconstruction demonstrating qualitative relationships (or lack thereof) between neurocranium geometry and conservation status.

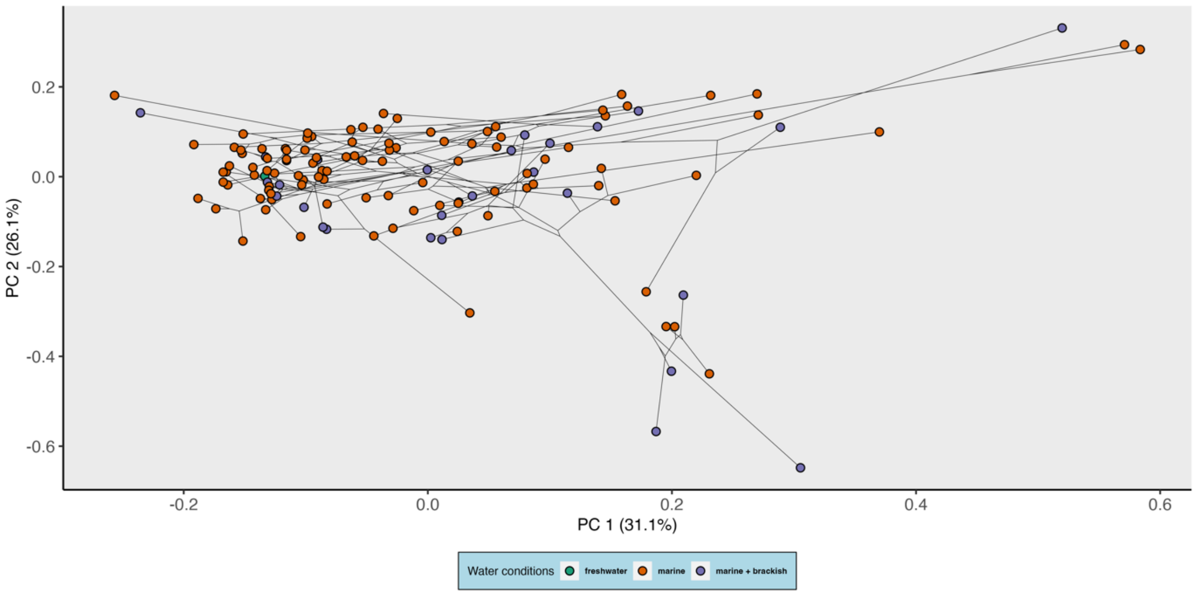

Supplementary Figure S7: phylomorphospace reconstruction demonstrating qualitative relationships (or lack thereof) between neurocranium geometry and water conditions.

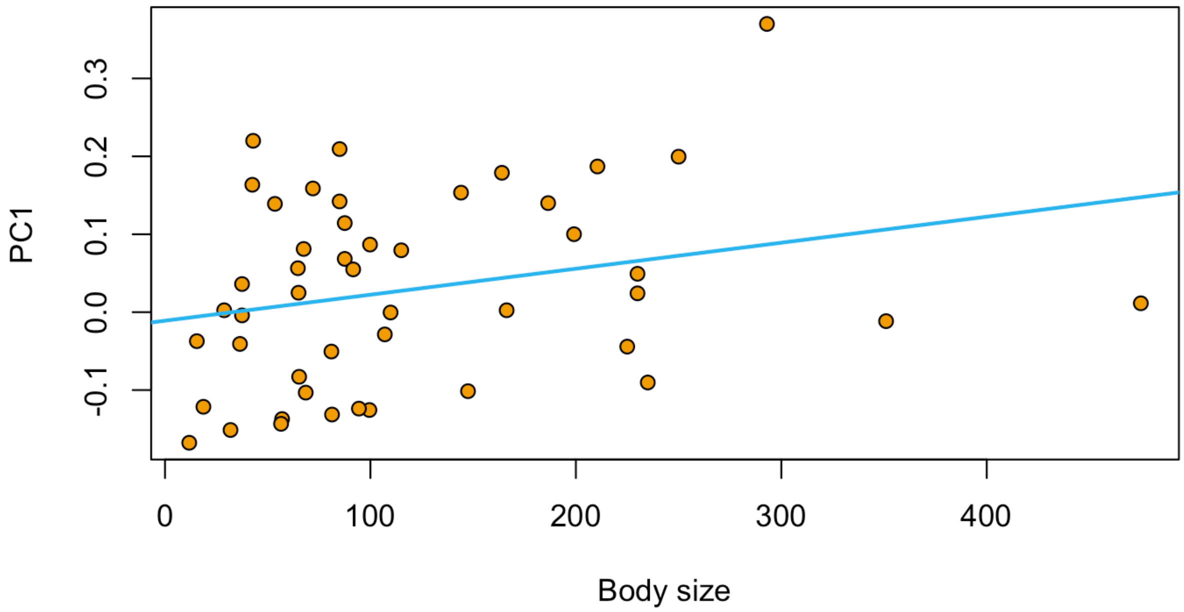

Supplementary Figure S8: PGLS plot displaying correlation between body size and PC1. Note that significant relationships were only found between those principal components and those covariates indicated as significant in Table 1.

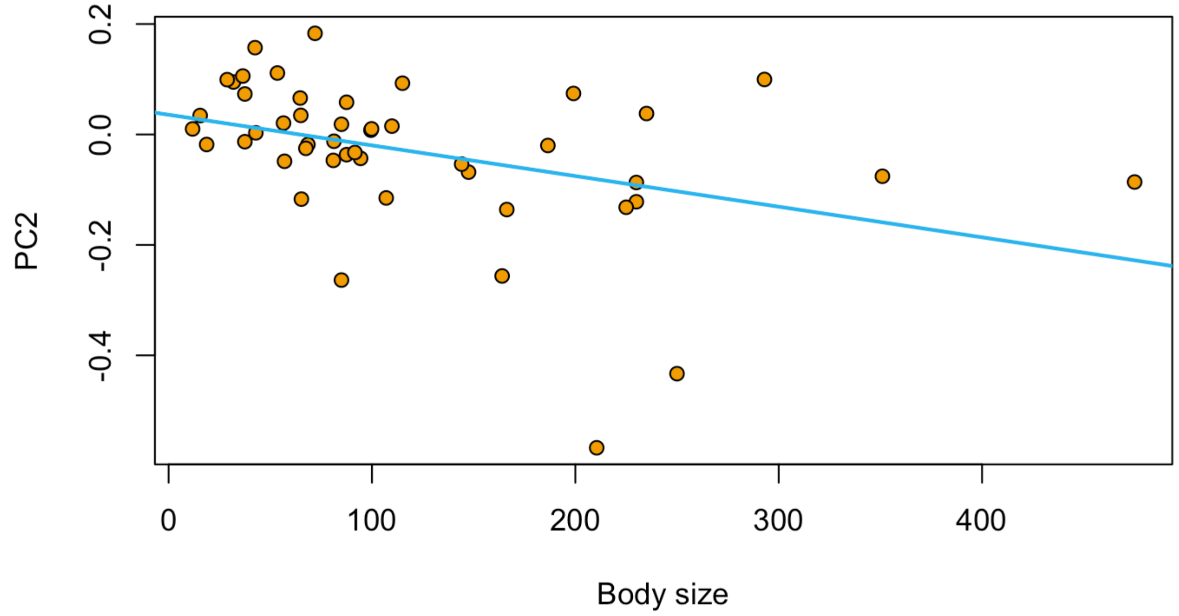

Supplementary Figure S9: PGLS plot displaying correlation between body size and PC2. Note that significant relationships were only found between those principal components and those covariates indicated as significant in Table 1.

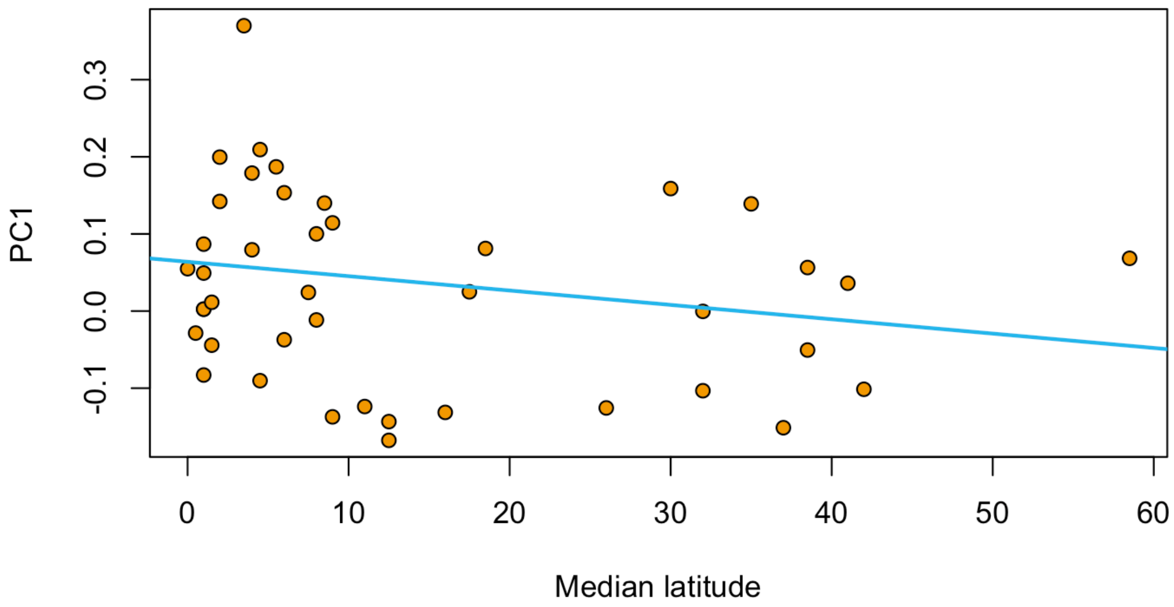

Supplementary Figure S10: PGLS plot displaying correlation between median latitude and PC1. Note that significant relationships were only found between those principal components and those covariates indicated as significant in Table 1.

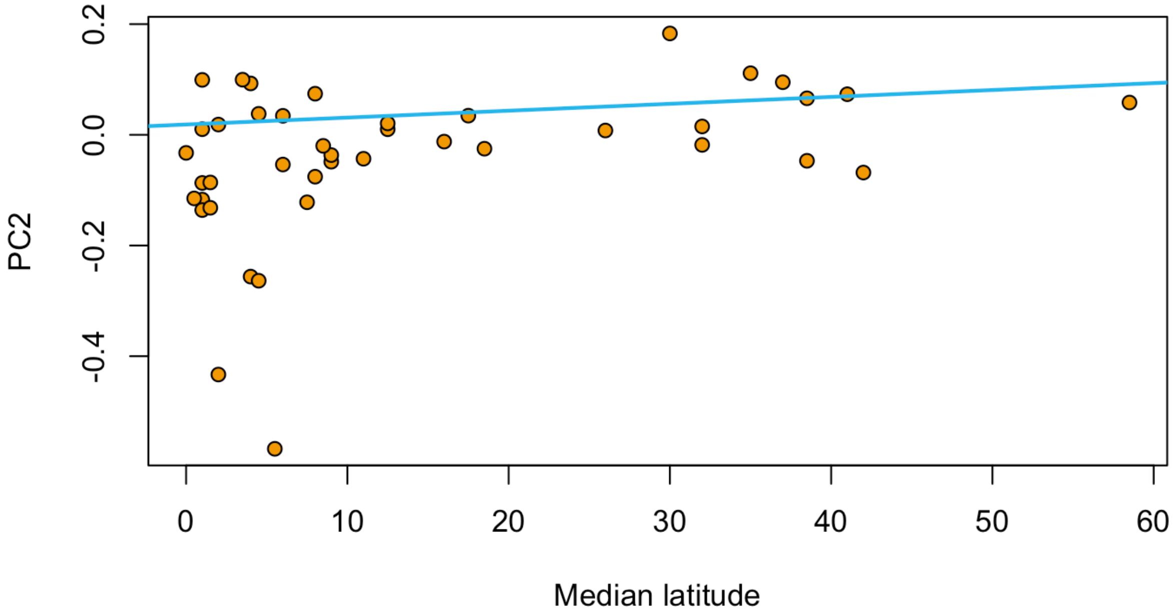

Supplementary Figure S11: PGLS plot displaying correlation between median latitude and PC2. Note that significant relationships were only found between those principal components and those

covariates indicated as significant in Table 1.

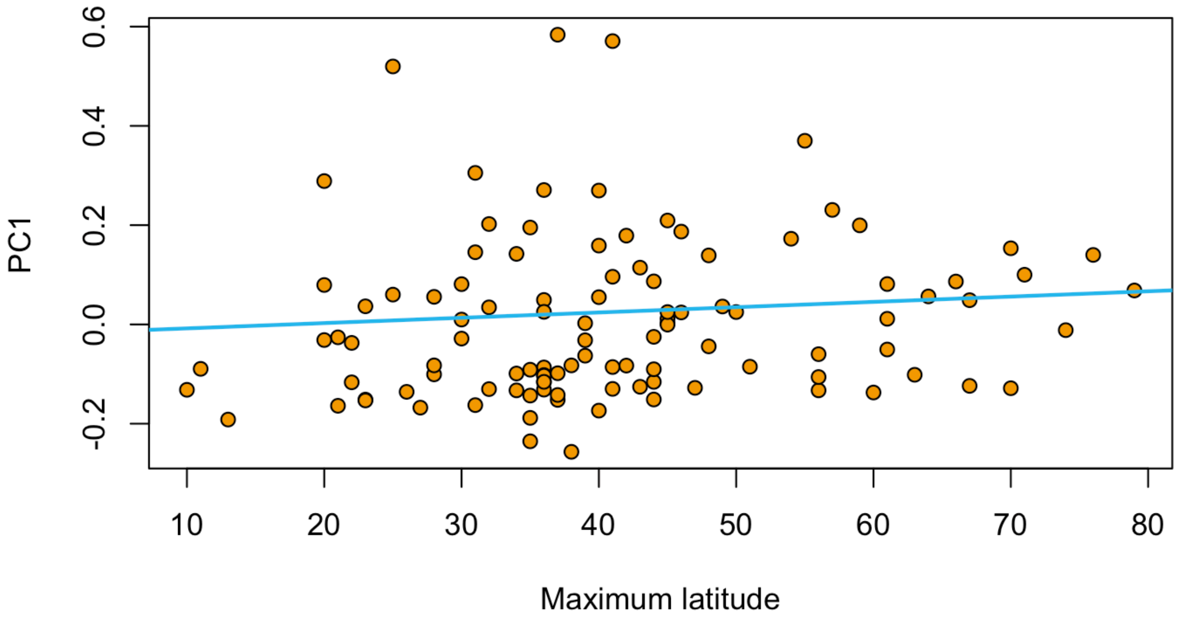

Supplementary Figure S12: PGLS plot displaying correlation between maximum latitude and PC1. Note that significant relationships were only found between those principal components and those covariates indicated as significant in Table 1.

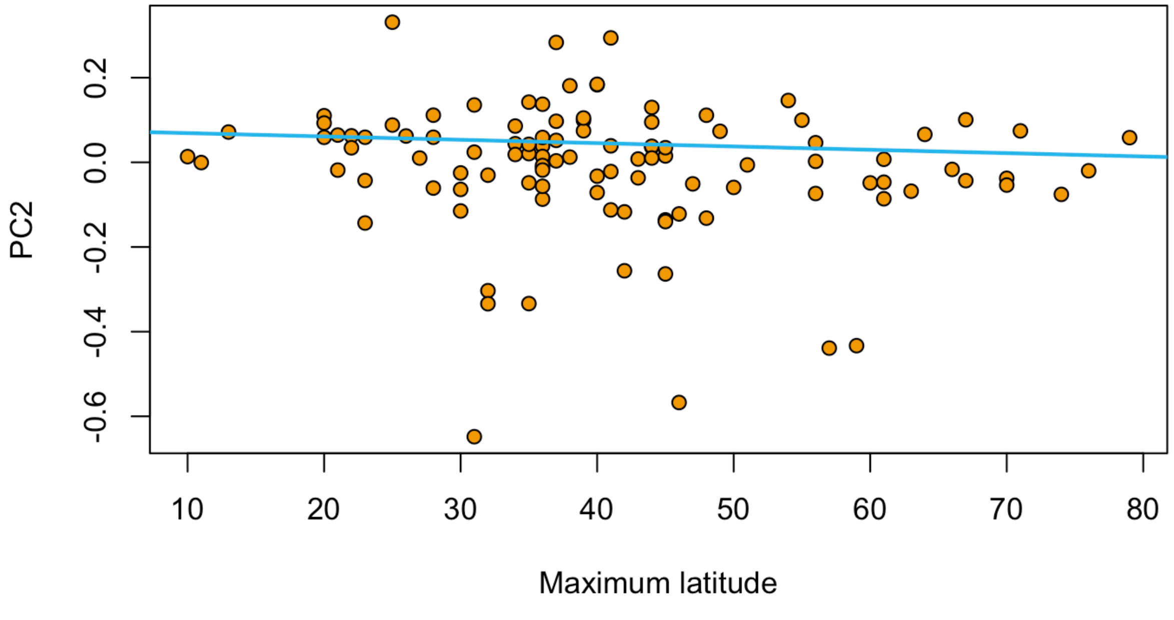

Supplementary Figure S13: PGLS plot displaying correlation between maximum latitude and PC2. Note that significant relationships were only found between those principal components and those

covariates indicated as significant in Table 1.

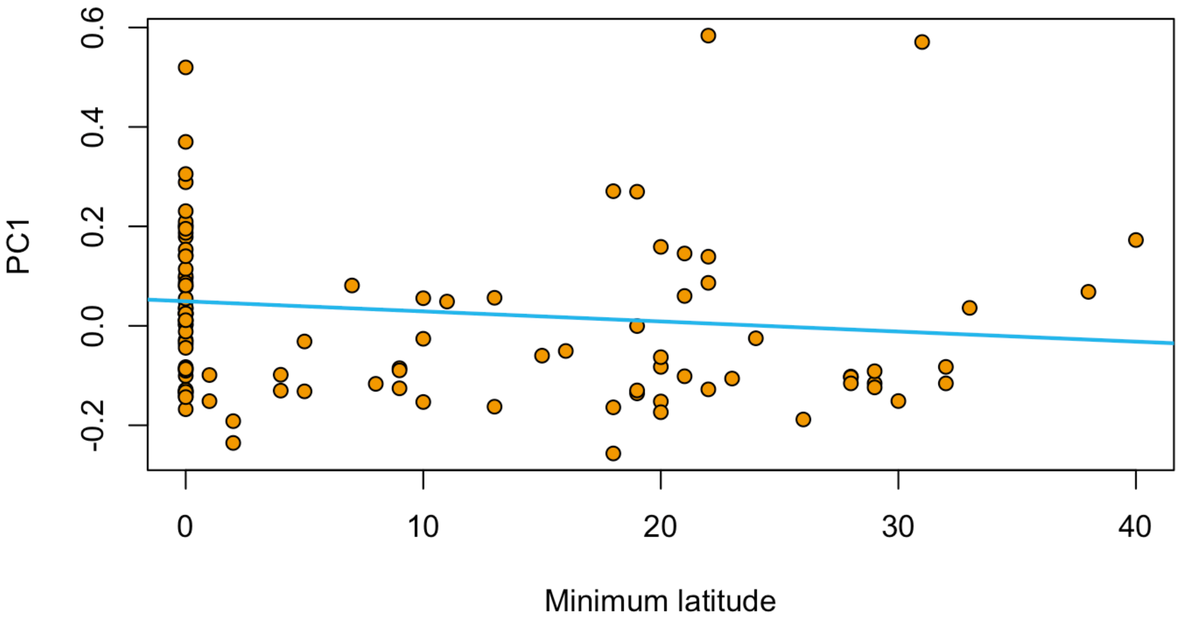

Supplementary Figure S14: PGLS plot displaying correlation between minimum latitude and PC1. Note that significant relationships were only found between those principal components and those covariates indicated as significant in Table 1.

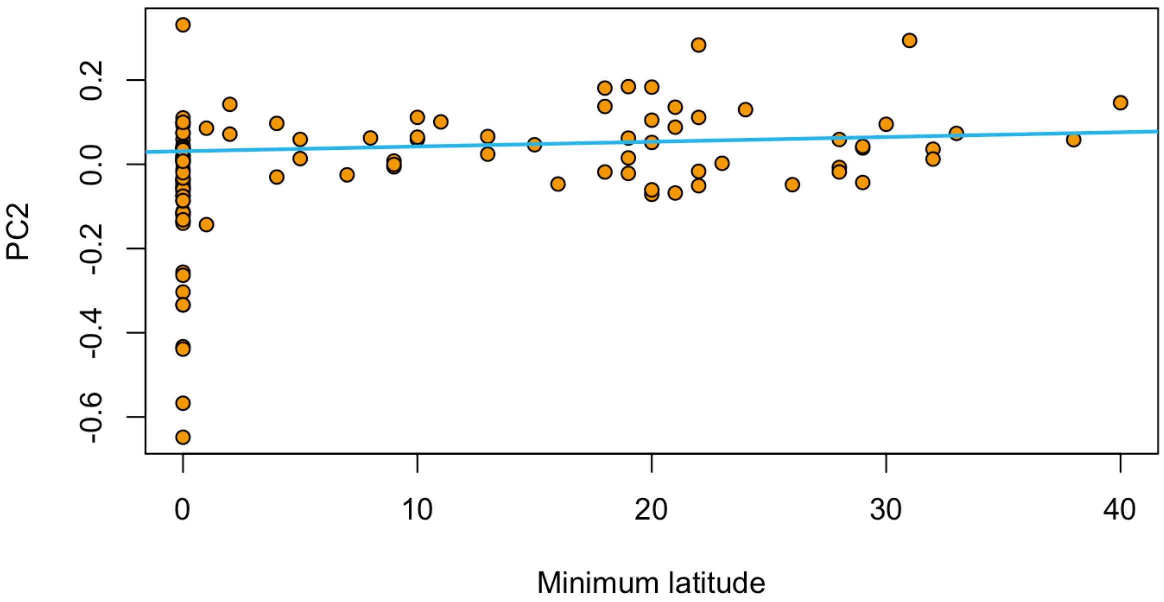

Supplementary Figure S15: PGLS plot displaying correlation between minimum latitude and PC2. Note that significant relationships were only found between those principal components and those

covariates indicated as significant in Table 1.

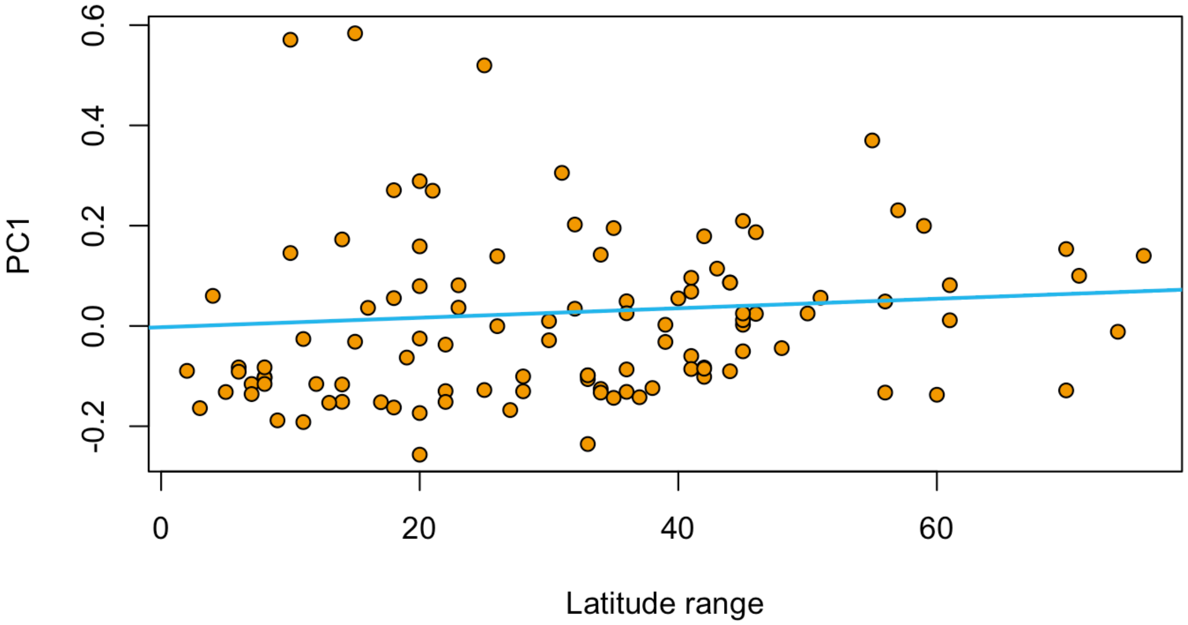

Supplementary Figure S16: PGLS plot displaying correlation between latitude range and PC1. Note that significant relationships were only found between those principal components and those covariates indicated as significant in Table 1.

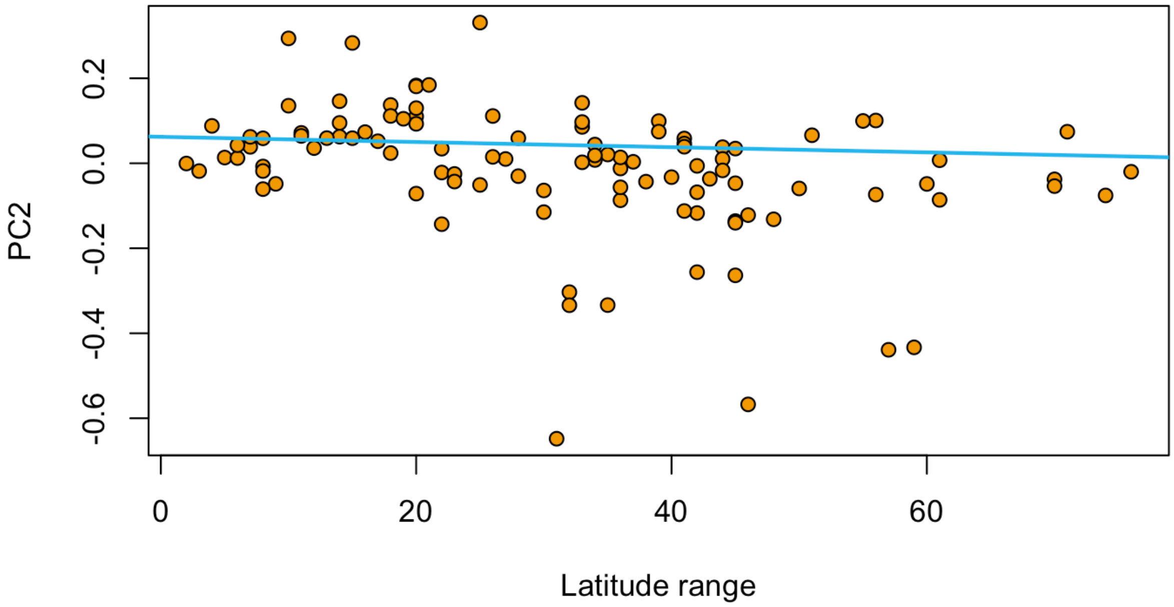

Supplementary Figure S17: PGLS plot displaying correlation between latitude range and PC2. Note that significant relationships were only found between those principal components and those

covariates indicated as significant in Table 1.

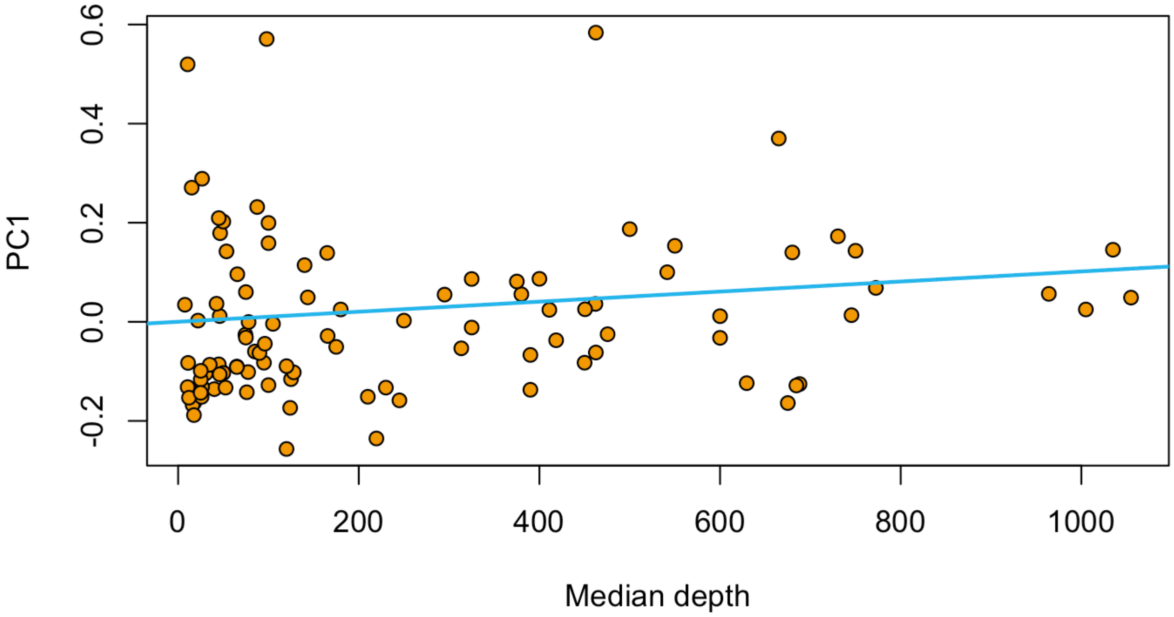

Supplementary Figure S18: PGLS plot displaying correlation between median depth and PC1. Note that significant relationships were only found between those principal components and those covariates indicated as significant in Table 1.

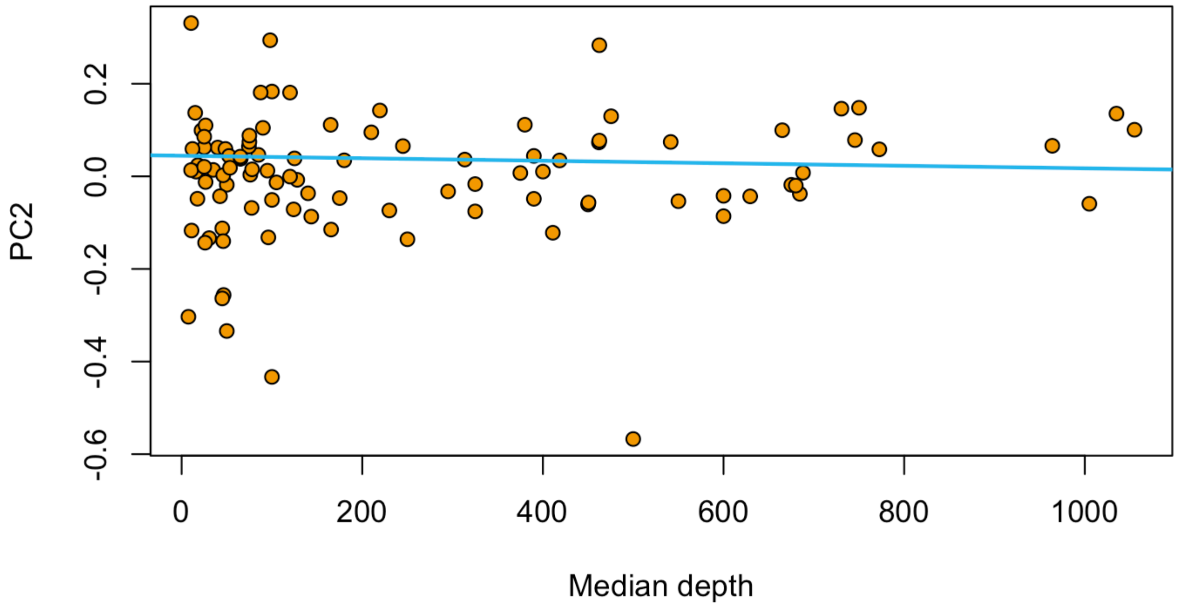

Supplementary Figure S19: PGLS plot displaying correlation between median depth and PC2. Note that significant relationships were only found between those principal components and those

covariates indicated as significant in Table 1.

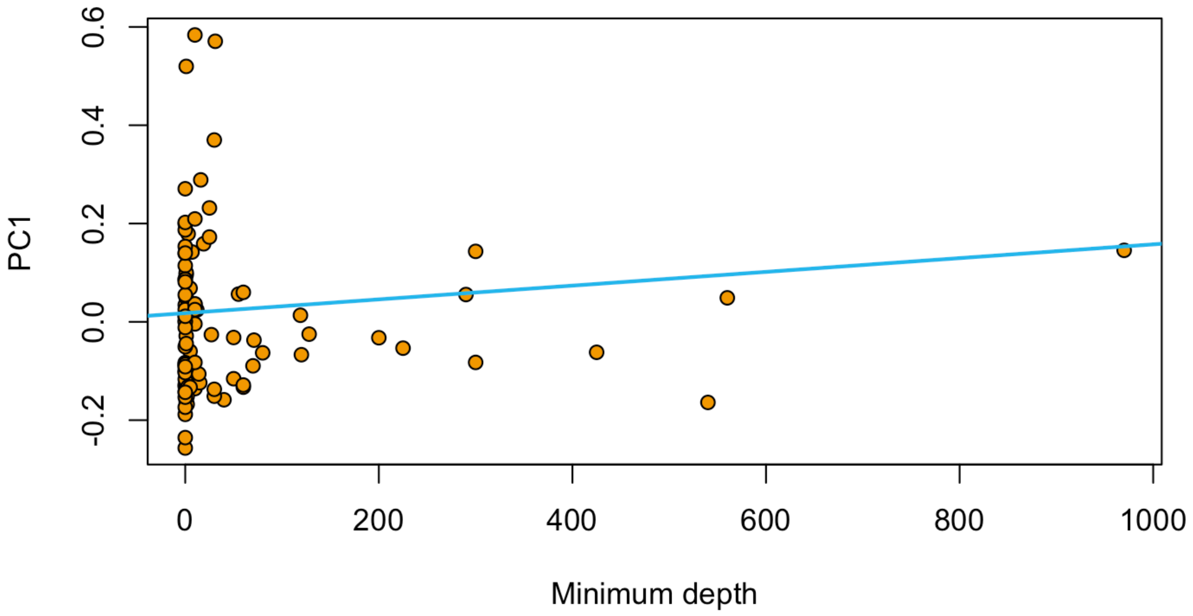

Supplementary Figure S20: PGLS plot displaying correlation between minimum depth and PC1. Note that significant relationships were only found between those principal components and those covariates indicated as significant in Table 1.

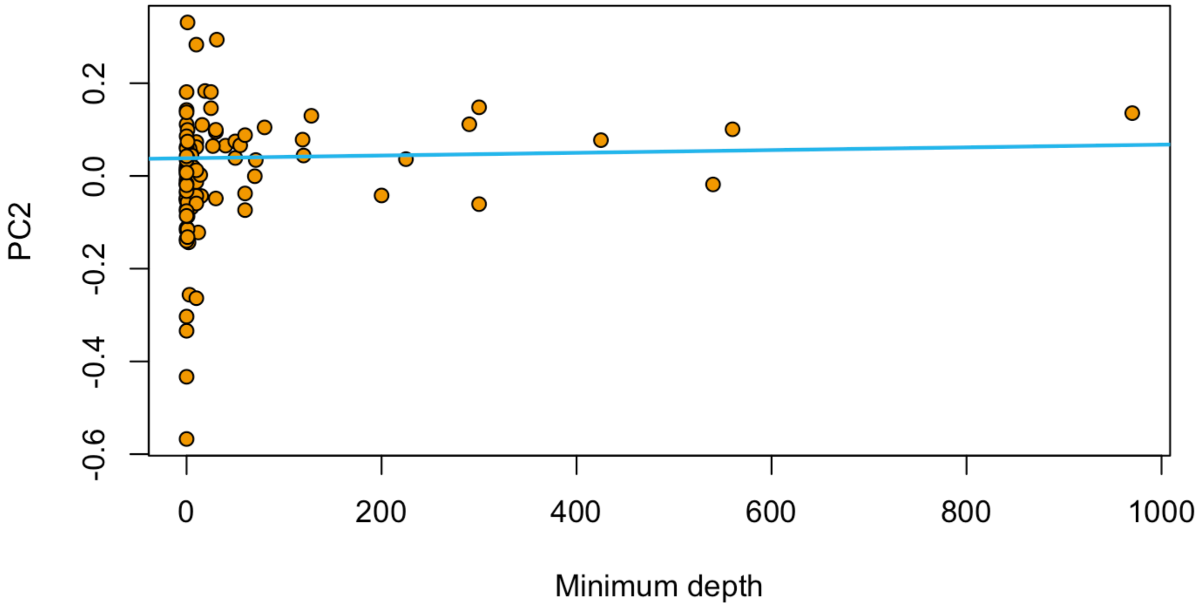

Supplementary Figure S21: PGLS plot displaying correlation between minimum depth and PC2. Note that significant relationships were only found between those principal components and those

covariates indicated as significant in Table 1.

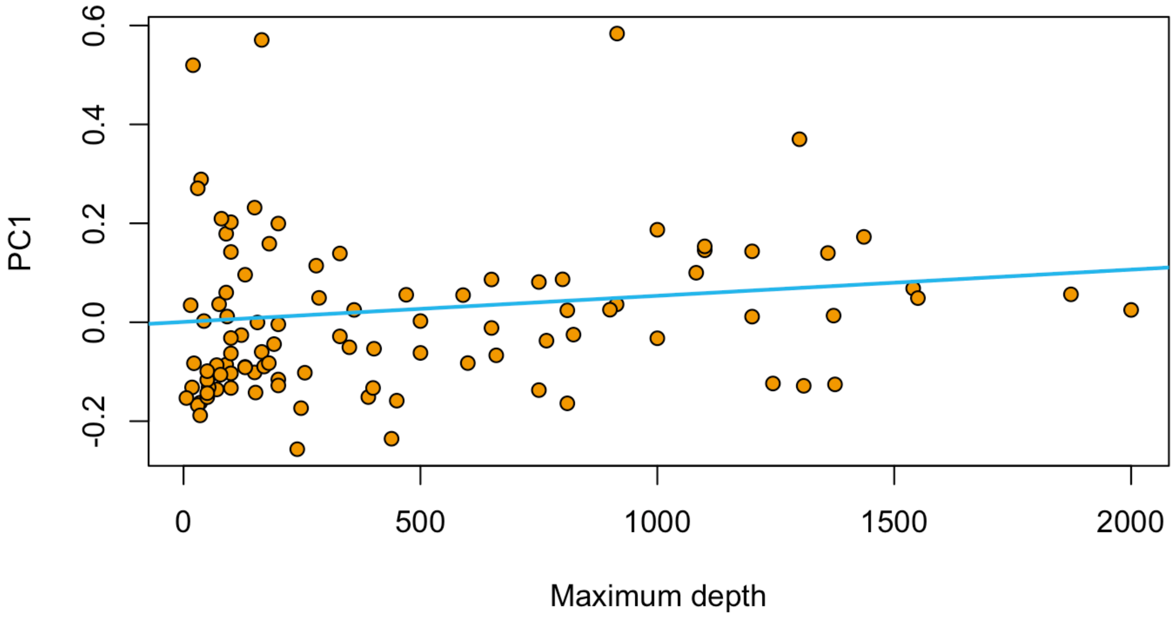

Supplementary Figure S22: PGLS plot displaying correlation between maximum depth and PC1. Note that significant relationships were only found between those principal components and those covariates indicated as significant in Table 1.

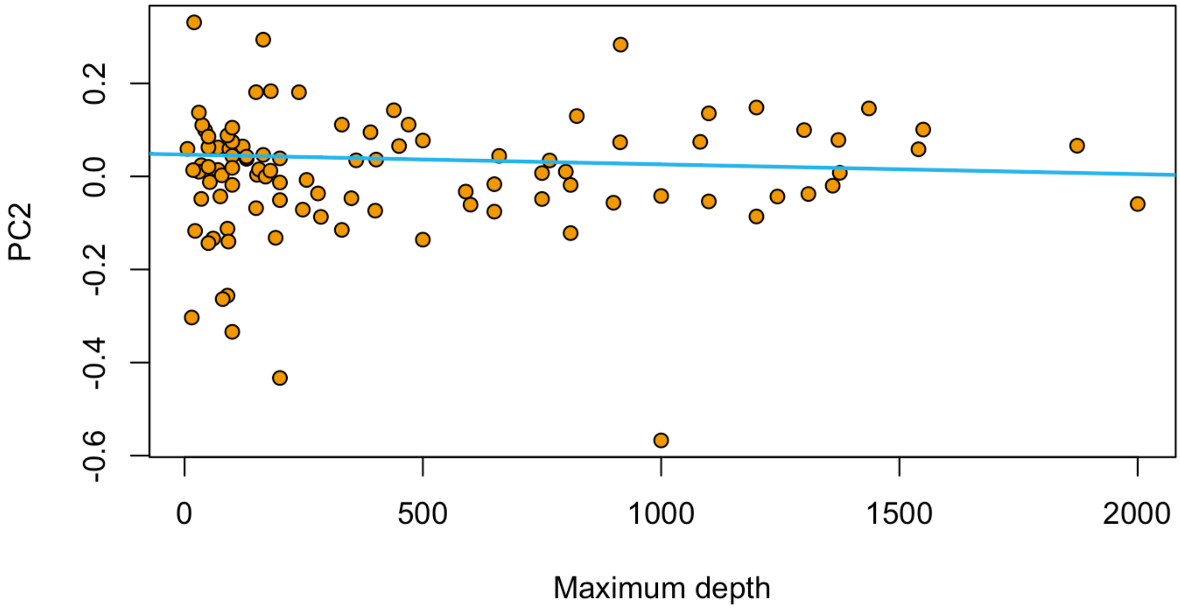

Supplementary Figure S23: PGLS plot displaying correlation between maximum depth and PC2. Note that significant relationships were only found between those principal components and those covariates indicated as significant in Table 1.

.

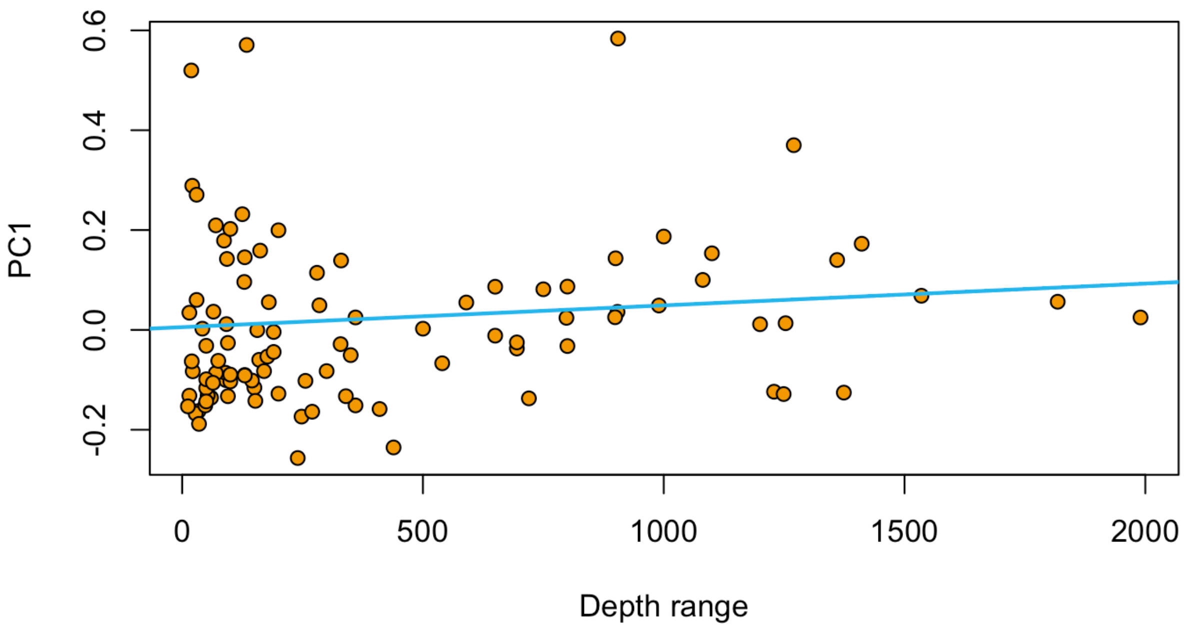

Supplementary Figure S24: PGLS plot displaying correlation between depth range and PC1. Note that significant relationships were only found between those principal components and those covariates indicated as significant in Table 1.

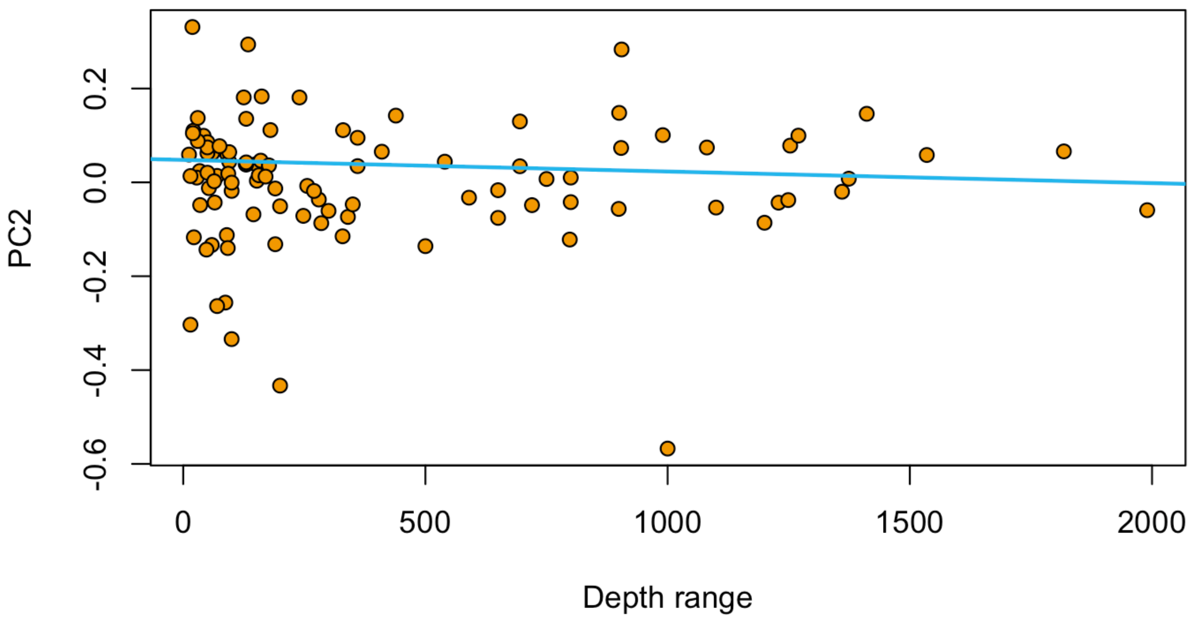

Supplementary Figure S25: PGLS plot displaying correlation between depth range and PC2. Note that significant relationships were only found between those principal components and those

covariates indicated as significant in Table 1.

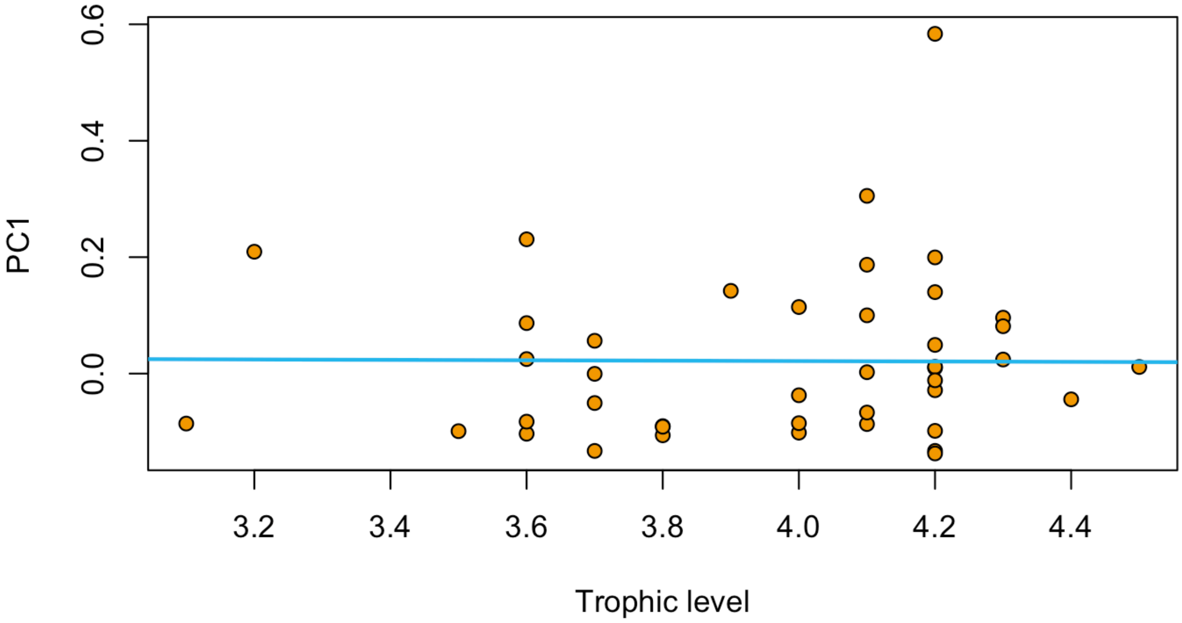

Supplementary Figure S26: PGLS plot displaying correlation between trophic level and PC1. Note that significant relationships were only found between those principal components and those covariates indicated as significant in Table 1.

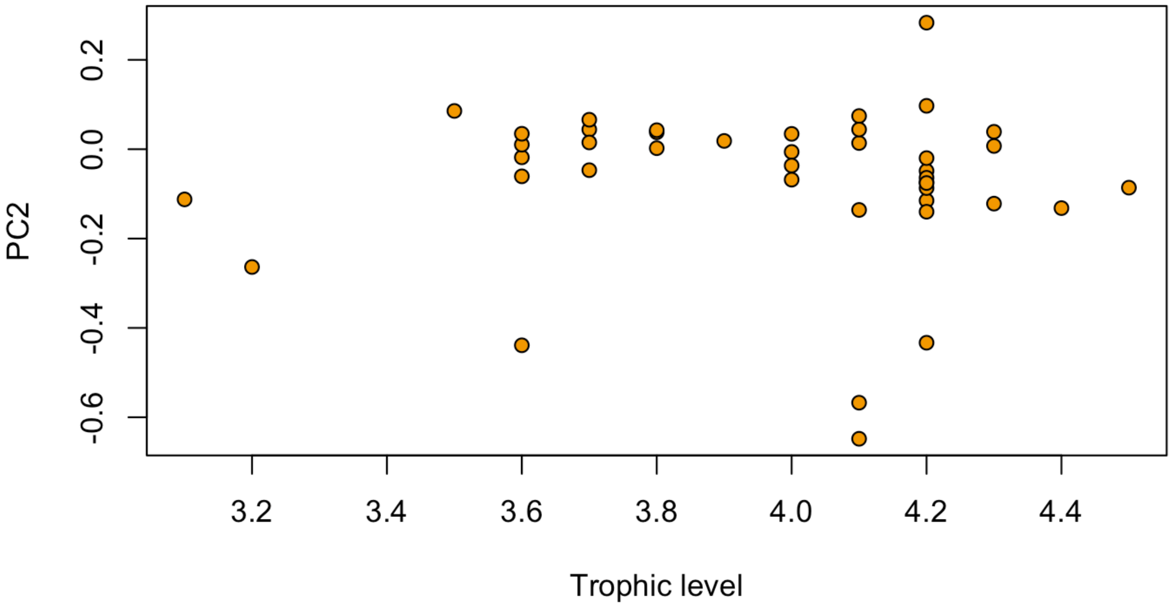

Supplementary Figure S27: PGLS plot displaying correlation between trophic level and PC2. Note that significant relationships were only found between those principal components and those covariates indicated as significant in Table 1.
